# CRISPR-associated type V proteins as a tool for controlling mRNA stability in *S. cerevisiae* synthetic gene circuits

**DOI:** 10.1093/nar/gkac1270

**Published:** 2023-01-18

**Authors:** Lifang Yu, Mario Andrea Marchisio

**Affiliations:** School of Pharmaceutical Science and Technology, Tianjin University, 92 Weijin Road, 300072 Tianjin, China; School of Pharmaceutical Science and Technology, Tianjin University, 92 Weijin Road, 300072 Tianjin, China

## Abstract

Type V-A CRISPR-(d)Cas system has been used in multiplex genome editing and transcription regulation in both eukaryotes and prokaryotes. However, mRNA degradation through the endonuclease activity of Cas12a has never been studied. In this work, we present an efficient and powerful tool to induce mRNA degradation in the yeast *Saccharomyces cerevisiae* via the catalytic activity of (d)Cas12a on pre-crRNA structure. Our results point out that dFnCas12a, (d)LbCas12a, denAsCas12a and two variants (which carry either NLSs or NESs) perform significant mRNA degradation upon insertion of pre-crRNA fragments into the 5′- or 3′ UTR of the target mRNA. The tool worked well with two more Cas12 proteins—(d)MbCas12a and Casϕ2—whereas failed by using type VI LwaCas13a, which further highlights the great potential of type V-A Cas proteins in yeast. We applied our tool to the construction of Boolean NOT, NAND, and IMPLY gates, whose logic operations are fully based on the control of the degradation of the mRNA encoding for a reporter protein. Compared to other methods for the regulation of mRNA stability in yeast synthetic gene circuits (such as RNAi and riboswitches/ribozymes), our system is far easier to engineer and ensure very high performance.

## INTRODUCTION

The precise and efficient expression of genes in an organism depends on mRNA synthesis, modification, and degradation (1). Thus, protein concentration can be controlled by regulating mRNA stability and translation. To this aim, three tools have been employed so far: RNA-binding proteins, riboswitches/ribozymes and short antisense RNA sequences. Pumilio/FBF (PUF) proteins, for instance, bind the mRNA 3′ UTR and recruit factors that reduce mRNA stability, which results in mRNA degradation (2,3). Antisense RNA molecules, such as small interfering RNAs (siRNAs) and micro-RNAs (miRNAs), cause mRNA cleavage and degradation, or repress translation via complementary base pairing with the target mRNA (4–6). Riboswitches and ribozymes respond to chemicals and allow to induce, mainly, translational repression (7).

The CRISPR-Cas system, an RNA-based immune system in bacteria and archaea, has become a popular instrument for genome editing (8–10), disease diagnosis (11,12), and gene therapy (13). To date, two classes and six types of CRISPR-Cas systems are known. Class 1—which comprises type I, III and IV—demands the cooperation of several Cas proteins to degrade foreign nucleic acids, whereas Class 2—which gathers type II, V and VI—requires a single, multi-domain Cas protein (Cas9, Cas12 and Cas13, respectively). The formation of mature CRISPR RNA (crRNA) from precursor crRNA (pre-crRNA) takes place, mainly, in two different ways. One—adopted by the popular CRISPR-Cas9—exploits the RNase III endoribonuclease that cleaves the pre-crRNA in the presence of trans-activating crRNA (tracrRNA) molecules. The other is fully dependent on the sole Cas protein. For instance, type I Cas6, isolated from the archaeon *Pyrococcus furiosus*, and Csy4, from *Pseudomonas aeruginosa*, catalyze pre-crRNA cleavage upon recognition of hairpin structures—referred to as direct repeats—along the pre-crRNA (14–18).

Csy4 endoribonucleases activity has been utilized to carry out RNA engineering in prokaryotes (19). Furthermore, Borchardt *et al.* showed that Csy4 and its cognate hairpin induced robust mRNA degradation in mammalian cells when placing the hairpin sequence either in the 5′ UTR (untranslated region) or just after the START codon (20). Recently, multiplex genome editing in plants was realized via the co-expression of Cas9 and Csy4 and the synthesis of a hybrid pre-crRNA, where Csy4 hairpins were placed between two different Cas9 crRNAs. This tool produced much better performance than the single transcript unit system in which Cas9 and the crRNA were placed in a single expression cassette and crRNA was generated, by self-cleaving ribozymes, under the control of a polymerase II promoter (21,22).

Type V Cas12a protein was characterized over the past few years (23–26) and found application in genome editing and transcription regulation (the latter via its nuclease deficient variant—dCas12a) (8,27–32). Like Csy4, Cas12a possesses pre-crRNA processing ability (25). Thus, the main goal of this work is to make use of (d)Cas12a as an RNA regulator—similarly to Csy4—to control gene expression in *Saccharomyces cerevisiae*.

Here, we show that three bare (d)Cas12a proteins—dFnCas12a, (d)LbCas12a, and denAsCas12a—and their variants (where (d)Cas12as are fused to either 2xNLS—nuclear localization sequence—or 2xNES—nuclear export sequence) induce mRNA degradation effectively in *S. cerevisiae* upon insertion of their pre-crRNA sequences in the mRNA 5′- or 3′ UTR. In particular, 5′ UTR ensures higher mRNA degradation efficiency than 3′ UTR. We refer to this new synthetic mRNA degradation system as (d)Cas12a:pre-crRNA tool. The bare dFnCas12a hardly degrades mRNA. However, its action improves significantly upon fusion with NLS or NES fragments. Moreover, dFnCas12a clearly prefers its cognate pre-crRNA. denAsCas12a, in contrast, appears to induce mRNA degradation more effectively as a bare protein. The three configurations of dLbCas12a manifest a bias toward different pre-crRNAs and are all highly efficient in degrading the mRNA. Our results also point out that mRNA degradation caused by the (d)Cas12a:pre-crRNA tool does not scale with the concentration of the target mRNA. Besides these three dCas12a, we tested three more Cas proteins, i.e. dMbCas12a, Cas12j-2 (also called Casϕ2) and LwaCas13a. dMbCas12a (30) and Casϕ2 perform poorly in transcription regulation and genome editing but outperform LwaCas13a in mRNA degradation in yeast. Based on these results, we made use of the mRNA-degradation capability of dCas12a to construct, in *S. cerevisiae*, NOT gates that respond to galactose or copper. Furthermore, we realized NAND and IMPLY gates (the latter responsive to copper and methionine) by means of a split dLbCas12a system. Overall, our results show a new way to use type V CRISPR-Cas systems as bioengineering tools.

## MATERIALS AND METHODS

### Plasmid construction

All plasmids constructed in this work were realized via the Gibson isothermal assembly method (33). As backbones, we utilized the yeast-shuttle vectors pRSII40X (obtained from Addgene, a gift from Steven Haase (34)). All plasmids used in this work are shown in Supplementary Material Excel sheet 1.

#### Construction of plasmids expressing yEGFP

Transcription units for yEGFP expression were first designed by inserting direct repeat hairpin structures of Fn/LbCas12a proteins (in a variable number) into the 3′- or 5′ UTR. Then, the modified UTRs were synthesized by Genewiz Inc., Suzhou (China) and finally assembled, together with the other parts (promoter, yEGFP, and terminator), into a cut-open pRSII405 backbone via Gibson method. pRSII405 was digested with Acc65I (NEB-R0599S) and SacI-HF (NEB-R3156S). TUs containing As/LbCas12a or LwaCas13a repeat along the 5′ UTR or MbCas12a repeats/Casϕ2 repeats/random sequences in the 3′ UTR were constructed by first extending *yEGFP* via PCR and then via Gibson assembly.

#### Construction of plasmids expressing Ttlcc1

Initially, the acceptor vector pRSII405-pTEF2-1xFn_Rs -Xbal-yEGFP-CYC1t-SacI, which host the FnCas12a direct repeat on pTEF2 5′UTR, was digested with Xbal (NEB-R0145S) and SacI-HF. Then, the cut-open vector was assembled, via Gibson method, with a PCR product consisting of Ttlcc1-CYC1t. Ttlcc1 had been yeast codon-optimized and synthesized by Genewiz Inc., Suzhou (China).

#### Construction of plasmids expressing type V (d)Cas proteins ((d)CasPs)

Plasmids containing yeast codon-optimized (d)CasP proteins had been previously synthesized by Genewiz Inc., Suzhou (China). From these plasmids, we PCRed out fragments of the form BamHI-(d)CasP-XhoI (where (d)CasP could be: dFnCas12a, (d)LbCas12a, denAsCas12a, dMbCas12a, and Casϕ2). These fragments were incorporated, via Gibson method, into acceptor vectors—realized in our lab—digested with BamHI (NEB-R0136V) and XhoI (NEB-R0146V). Our acceptor vectors had the form: pRSII406-pGPD/pCUP1/pGAL1/pMET25-ATG-(NLS/NES)-GS-HIStag-GS-BamHI-sp-Xhol-GS-(NLS/NES)-TAA-CYC1t, where ‘sp’ is a short, random sequence. They could contain two NLSs, two NESs, or none of them.

All PCRs we carried out were touchdown PCRs. They required Q5 High-Fidelity DNA Polymerase (NEB-M0491S). PCR products were collected and purified by using AxyPrep DNA extraction kit (Axigen-AP-GX-250). DNA fragments were added, in equimolar amount, to 15 μl Gibson mixture. Isothermal assembly took one hour at 50°C in a Thermal cycler machine. Finally, *E. coli* competent cells (DH5α, Life Technology—18263–012) were transformed with 5 μl of isothermal assembly products. The specific transformation steps are described in (30).

### Yeast transformation

We used as a chassis for our circuits the *S. cerevisiae* strain CEN.PK2-1C (MATa; his3Δ1; leu2-3_112; ura3-52; trp1-289; MAL2-8c; SUC2), Euroscarf-30000A (Johann Wolfgang Goethe University, Frankfurt, Germany)—termed byMM584. New strains based on byMM584 were created by integrating plasmids into its genome. Yeast transformation was carried out according to the standard lithium acetate – thermal shock method (35). Transformed yeast cells grew for 2 to 4 days at 30°C on plates (2% agar, 2% glucose) containing synthetic selective media. All yeast strains engineered in this work are listed in Supplementary Material Excel sheet 2.

### Cell culture and media

We used YPD rich medium (2% bacto-peptone, 1% yeast extract, adenine hemisulfate, and 2% glucose) for original strain recovery and yeast transformation. A synthetic defined complete medium (SDC) supplemented with either 2% glucose or 2% galactose was used for growing cells before FACS experiments. As for Ttlcc1 selection plates (36), we prepared SDC plates containing 1 mM CuSO4 (Innochem-Cat: 7758-98-7) and 0.5 mM ABTS (CAS number: 9003-99-0). SDC minus copper (SDC-YNB_Cu) was made by replacing YNB with ‘YNB without copper material’ (Coolaber-Cat# PM2070-Cu-250g). Copper sulfate was used to carry out copper titration curves. A 10 mM methionine stock solution was prepared by mixing 374 mg of methionine (CAS number: 63-68-3) with 250 ml of SDC (without methionine) solution. It was used in the methionine titration curve and the tests on the IMPLY gates.

### Ttlcc1 characterization

Yeast cells were grown overnight on YPD plates. Afterwards, cells were picked with a sterile loop and dissolved in 5 ml of SDC fresh solution to reach OD_600_ equal to approximately 2.0. 50 μl of cell solutions were then poured on Ttlcc1 selection plates (see above). The plates were placed into an incubator at 30°C and photos were taken every 12 h.

### FACS measurement

Strains were cultured at 30°C and 240 RPM for approximately 18 h—in order to reach the steady state—in 2 ml SDC (supplemented with 2% glucose or galactose) or SDC supplied with different concentrations of copper/methionine. 20 μl of cell solution were then diluted into 300 μl ddH2O before any FACS measurement. We measured the fluorescence intensity from our strains (due to yEGFP) with a BD FACSVerse machine (laser 488 nm-FITC filter 527/32 nm). In order to have reliable measurements the BD FACSVerse should have passed the Performance Quality Control (PQC). Moreover, each PQC was followed by fluorescent beads (BD FACSuite CS&T Research beads 650621) measurement to adjust the FITC voltage. If the relative difference between the values of the bead peak in two independent experiments was not bigger than 5%, then the machine conditions had not changed considerably during these experiments and the collected data was reliable (see the diagram in Supplementary Figure SMM1). All FACS files were analyzed via the flowcore R-Bioconductor package (37). Ten thousand cells were collected during each measurement. Fluorescence mean values were calculated on at least three independent experiments. We used two-sided Welch's *t* test to do the statistical analysis (all fluorescent data collected via FACS is available in Supplementary Material Excel sheet 3).

### Tecan measurement

Strains used in Casϕ2- and LbCas12a-driven genome editing were measured on a Tecan machine Infinite M200 PRO (see Supplementary Material Excel sheet 4 and 5). Strains were cultured in 2 ml SDC for 18 h (30°C, 240 RPM) to reach the steady state. Yeast cells were then collected via 1 min centrifugation at max speed. After removing the supernatant, yeast cells were resuspended in 1 ml ddH_2_O and then transferred to transparent and black 96-well plates (200 μl for each well). The former type of plate was used to measure OD_600_, the latter to detect the overall fluorescence from a cell population (excitation wavelength: 476 nm; emission wavelength: 512 nm). The average fluorescence intensity (FI) was calculated as follows (the background strain, here, is byMM584):

$$\begin{eqnarray*} {\rm Average\ FI}(_{\rm sample}) &=& {\rm FI}(_{\rm sample})/{\rm OD}(_{\rm sample}) \nonumber \\ &&-\, {\rm FI}(_{\rm background})/{\rm OD}(_{\rm background}) \end{eqnarray*}$$

### Growth curve

Yeast strains, taken from cryostock tubes, were grown overnight in 5 ml SDC solution at 30°C and 240 RPM. In the morning, the optical density (OD_600_) of the strain solutions was measured with an Eppendorf BioPhotometer apparatus. Afterwards, cells were diluted into 30 ml of fresh medium to reach OD_600_ between 0.1 and 0.2. Strains were, then, cultured for 18 h during which OD_600_ was measured every two h (see Supplementary Material Excel sheet 6).

## RESULTS AND DISCUSSION

### dCasP degrades mRNA robustly after inserting pre-crRNA sequences into the mRNA 3′ UTR

**Figure 1.**
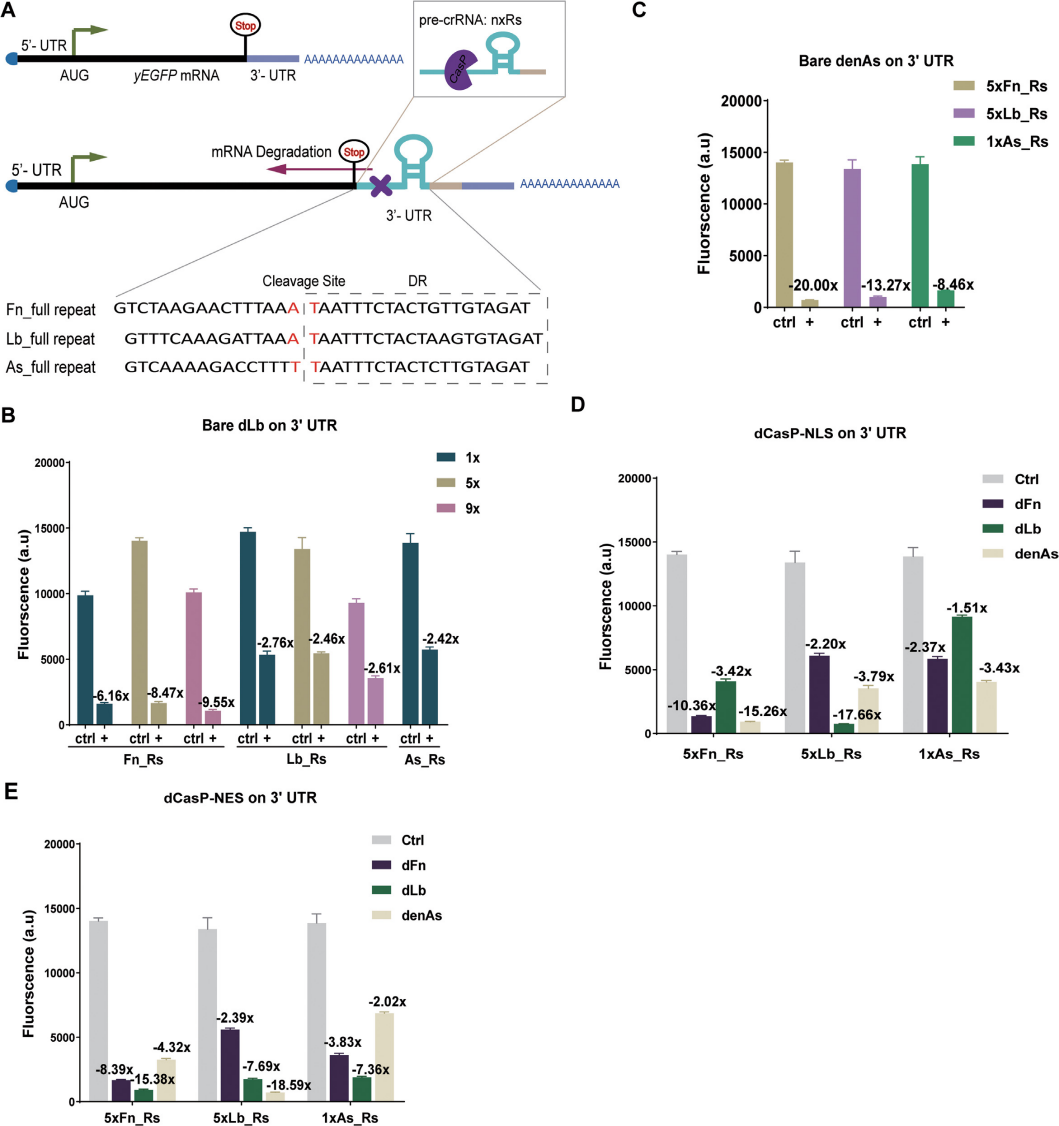
dCasP mediates yEGFP mRNA degradation by cleaving the pre-crRNA repeats placed along the mRNA 3′ UTR. (**A**) mRNA degradation mechanism. A mature mRNA consists of the 5′ cap (blue circle) followed by the 5′ UTR and the yEGFP-coding mRNA (black rectangle); the STOP codon; the 3′ UTR; and, finally, the poly(A) tail. dCasP pre-crRNA sequences (i.e. one or multiple repeat—spacer motifs) are placed downstream of the STOP codon along the 3′ UTR. dCasP recognizes the hairpin structure of the repeat and cuts the pre-crRNA in its proximity, triggering, in this way, mRNA degradation. The full repeat sequence of three Cas12a proteins is here reported. (B–E) mRNA degradation efficiency of dCasP. 1x, 5x and 9x refer to the number of Rs in the pre-crRNA. ‘Ctrl’ represents the control circuit in which dCasP is missing. Values on different columns represent the ratio between the green fluorescence intensity in the control and the dCasP:pre-crRNA-containing strains. (**B**) and (**C**) refer to the mRNA degradation caused by the yeast codon-optimized bare dLbCas12a and denAsCas12a, respectively. The results in (**D**) and (**E**) represent the effects of mRNA degradation induced by the two dCasP variants containing either 2xNLS or 2xNES. Mean fluorescence levels were calculated from at least three independent experiments (FACS measurements), i.e. carried out in different days.

We reasoned that mRNA in *S. cerevisiae* could be degraded by type V (d)Cas proteins (referred to as (d)CasP throughout this work) if we incorporated dCasP pre-crRNA sequences into the UTRs of the target mRNA. To verify our hypothesis, we used initially two dCasP proteins in their original, bacterial nucleotidic sequences—bac_dFnCas12a (abbreviated as bac_dFn) and bac_dLbCas12a (bac_dLb)—and analyzed their action on the mRNA of the yeast enhanced green fluorescent protein (yEGFP) modified with the insertion of dCasP pre-crRNA into the 3′ UTR (see Supplementary Figure S1A). The pre-crRNA sequences used in this work contain a variable number (n) of direct repeat—spacer (Rs) structures, namely: one, five, or nine Rss (denoted as 1xRs, 5xRs and 9xRs, respectively). Direct repeats are identical along a given pre-crRNA, whereas spacers can be different and are selected from bacterial genomes to avoid OFF-target effects (38) due to the activity of the dCasP:crRNA complex (see also ‘OFF-target Effect Analysis’ in Supplementary Material and the relating Supplementary Material-transcriptome file). When expressed in yeast, dCasP targets and cleaves its pre-crRNA sequences, which triggers mRNA decay (see Figure 1A). This causes a reduction in the yEGFP expression level. Since the *yEGFP* gene was placed downstream of a rather weak minimal *CYC1* promoter (pCYC1min), we considered, at the beginning, only strains where the TU pCYC1min-yEGFP-nxRs-CYC1t (also referred to as modified TU1, see Supplementary Figure S1B) was integrated twice. In this way, we could measure fluorescence levels high enough to estimate the effects of mRNA degradation. In each experiment, mRNA-degradation circuits were compared to a control one where the dCasP expression cassette was absent.

As shown in Supplementary Figure S1C, bac_dFn and bac_dLb cleaved both cognate and non-cognate pre-crRNA, in a rather inefficient way, though.

To enhance mRNA degradation, we replaced bac_dCasPs with their yeast codon-optimized versions, yo_dCasPs (for the sake of simplicity, from now on we will refer to any yo_(d)CasP simply as (d)CasP). We also considered a third dCasP, denAsCas12a (denAs), whose performance as a transcription factor in *S. cerevisiae* is comparable to that of dLbCas12a (dLb) (30). Surprisingly, dLb turned out to be more effective on Fn_Rs rather than on its cognate pre-crRNA. Moreover, fluorescence inhibition due to Fn_Rs cleavage by dLb increased with the number of repeat-spacers (up to 9.55-fold on 9xFn_Rs, see Figure 1B). denAs also performed better on non-cognate pre-crRNAs (20.00-fold on 5xFn_Rs, see Figure 1C). It should be noted, though, that we used as a target only a single (and no multiple) As_Rs. As shown in Supplementary Figure S1D, dFn failed to improve bac_dFn results.

To further enhance mRNA degradation, we built two variants of each dCasP via the fusion of either two nuclear localization sequences (NLSs) or two nuclear export sequences (NESs) (dCasP-NLS/NES)—see Supplementary Figure S1A. dLb–NLS improved drastically the cleavage of its cognate pre-crRNA, by reaching a 17.66-fold fluorescence decrease on 5xLb_Rs. In contrast, its action on non-cognate pre-crRNAs was less effective than that of the bare dLb. denAs-NLS did not achieve the same inhibition levels as the bare denAs. However, it showed a remarkable 15.26-fold of fluorescence reduction on 5xFn_Rs (see Figure 1D and Supplementary Figure S1E). The action of dFn-NLS was noteworthy on its cognate pre-crRNA only (10.36-fold fluorescence inhibition on 5xFn_Rs—see Figure 1D). Moreover, we checked mRNA degradation mediated by LbCas12a-NLS (that maintained DNA cleavage activity—see Supplementary Figure S1E-F). Similar to dLb-NLS, the highest fluorescence inhibition was registered on 5xLb_Rs (14.45-fold).

dCasP fused with NES fragments was tested only on 1xAs_Rs and 5xLb/Fn_Rs configurations as shown in Figure 1E. Similar to dLb, dLb-NES carried out a strong mRNA degradation by acting on a non-cognate pre-crRNA (best inhibition of fluorescence: 15.38-fold on 5xFn_Rs), whereas it was much less effective than dLb-NLS on the cognate pre-crRNA. denAs-NES showed a weak affinity towards both 5xFn_Rs and the cognate pre-crRNA but a strong one for 5xLb_Rs that led to a 18.59-fold fluorescence inhibition. As for dFn-NES, its overall performance was like that of dFn-NLS, with the highest fluorescence inhibition obtained on the cognate pre-crRNA.

To make our results more solid, we constructed a second control group in which the sequences of pre-crRNA were replaced with random bases to avoid mRNA degradation (see Supplementary Figure S1G).

On the whole, our results highlight that three yeast codon-optimized dCasP (dFn, dLb and denAs) either bare or fused to 2xNLS/NES degrade mRNA efficiently in *S. cerevisiae* in the presence of pre-crRNA along the 3′ UTR of the target mRNA. dFn induced mRNA degradation only when fused to two NLSs or NESs and performed better by acting on its cognate pre-crRNA. In contrast, no matter the pre-crRNA, the bare denAs outperformed its two variants—except on 5xLb_Rs where denAs-NES achieved the highest fluorescence repression. The bare dLb and dLb-NES displayed their highest affinity towards the non-cognate sequence 5xFn_Rs, whereas dLb-NLS caused a remarkable fluorescence decrease when acting on 5xLb_Rs.

### dCasP degrades mRNA robustly upon insertion of pre-crRNA sequences into the mRNA 5′ UTR

**Figure 2.**
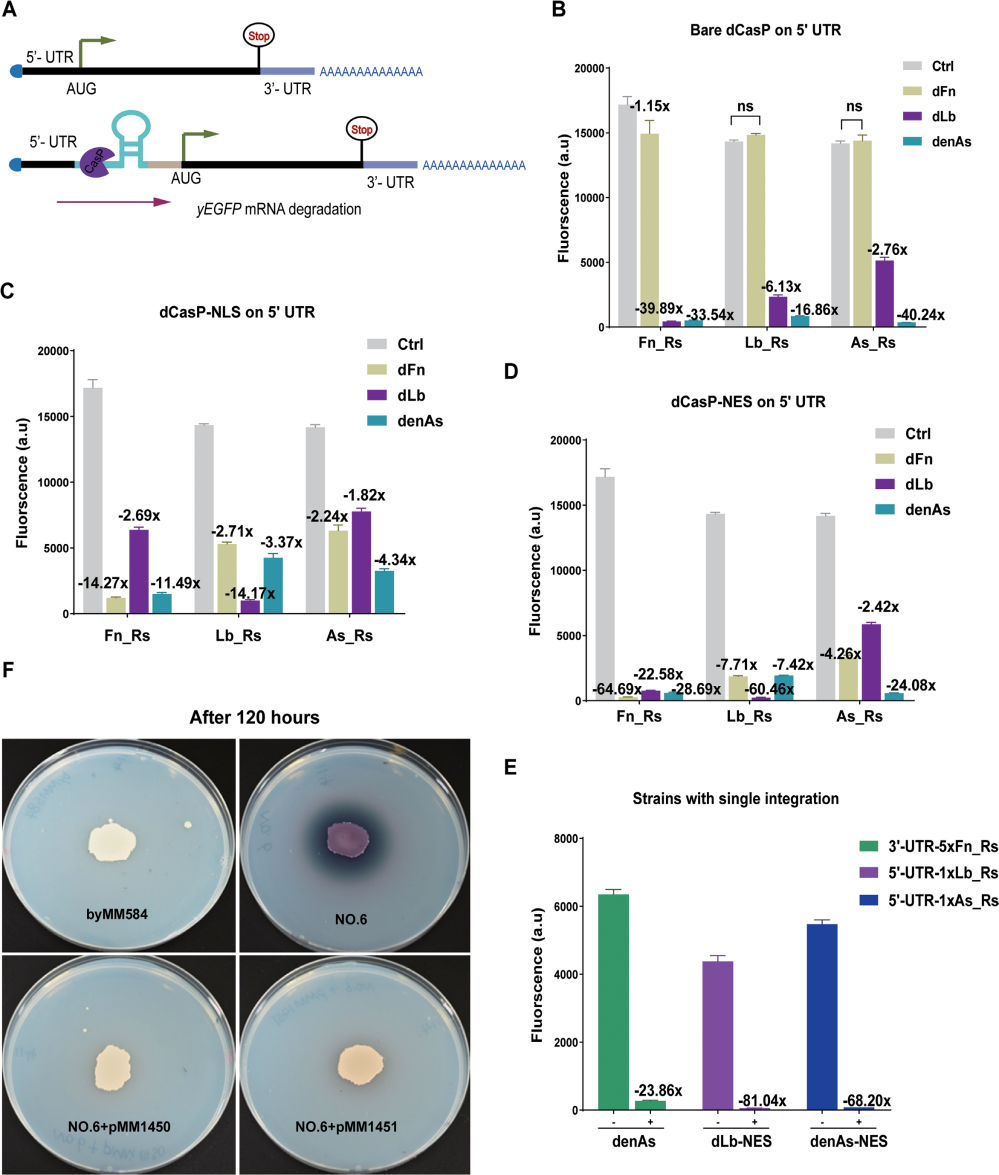
dCasP and its variants (dCasP-NLS and dCasP-NES) induce the degradation of the mRNA of a reporter protein by cleaving a pre-crRNA hairpin structure inserted into the 5′ UTR. (**A**) Circuit diagram. A pre-crRNA sequence, added upstream of the START codon of yEGFP, is recognized by dCasP. Once dCasP has bound the pre-crRNA, it cuts the Rs at the cleavage site, which triggers mRNA degradation. (**B–D**) Action of dCasP and its two variants on the mRNA of yEGFP. ‘Ctrl’ (control) is a strain that does not contain dCasP. The values on top of the columns represent the ratio between the fluorescence intensity of the control and the full-circuit-containing strains. (**E**) mRNA degradation efficiency of the strains where the plasmid carrying yEGFP was integrated only once in the yeast genome. (**F**) dFn-NES and denAs-NES mediated mRNA degradation of Ttlcc1 upon placing 1xFn_Rs upstream of ATG of *Ttlcc1* gene. byMM584 is the empty chassis. ‘NO.6’ indicates the strain that contains the pTEF2-1xFn_Rs-Ttlcc1-CYC1t expression cassette alone. ‘NO.6 + pMM1450/1451’ refer to the strains where either pMM1450 (dFn-NES) or pMM1451 (denAs-NES) was integrated into byMM584 together with ‘NO.6’. The four strains were grown for 120 h on SDC plates supplied with 1 mM CuSO_4_ and 0.5 mM ABTS. Each fluorescence level is the mean value from at least three independent experiments (FACS), i.e, carried out in different days. ‘ns’ stands for no statistically significant difference (P-value > 0.05; two-sided Welch's t test).

Next, we placed 1xRs pre-crRNA sequences along the 5′ UTR of TU2 (pTEF2-yEGFP-CYC1t—see Figure 2A and Supplementary Figure S2A) and selected strains where the modified TU2 was double-integrated. We tested dCasP (Figure 2B) and its two variants (dCasP-NLS and dCasP-NES—see Figure 2C, D) directly, since bac_dCasP failed to induce a considerable mRNA degradation when the pre-crRNA was located inside the 3′ UTR.

Consistent with the results in the previous section, the low affinity of the bare dFn towards every Rs prevented any significative pre-crRNA cleavage. However, dFn became ‘functional’ upon fusion to 2xNLS or 2xNES. Both dFn–NLS and dFn–NES accomplished fluorescence inhibition by acting on their cognate pre-crRNA (Fn_Rs): 14.20-fold (dFn-NLS) and an impressive 64.69-fold (dFn-NES). On the non-cognate pre-crRNAs, dFn-NES also appeared more effective than dFn-NLS.

dLb, in its bare configuration, induced higher yEGFP inhibition on the non-cognate Fn_Rs (39.89-fold) rather than on its own pre-crRNA (Lb_Rs, 6.13-fold), similar to the case of 3′ UTR. Upon fusion to 2xNLS and 2xNES, the action of dLb on Fn_Rs became less strong. dLb-NLS and dLb-NES displayed a clear preference towards their cognate pre-crRNA. The former provoked a 14.17-fold fluorescence reduction on Lb_Rs, the latter caused even a 60.46-fold repression in fluorescence. dLb-NES showed high affinity also towards Fn_Rs, whereas no configuration of dLb appeared particularly effective on As_Rs.

As for the bare denAs, it manifested higher affinity towards pre-crRNA sequences than its two variants, consistently with the trend observed on the 3′ UTR. Under the action of denAs, fluorescence decreased up to 40.24-fold (As_Rs) and 33.54-fold (Fn_Rs). High fluorescence reduction was caused also by denAs-NES on Fn_Rs (28.69-fold) and As_Rs (24.08-fold).

In 2017, Liang *et al.* obtained fluorescence inhibition, in plants, through the action of Csy4 on its corresponding repeat that was placed along the mRNA of the yEGFP (39). They found that fluorescence reduction due to mRNA degradation was directly proportional to the expression level of yEGFP. To check if the same phenomenon was present in our dCasP:pre-crRNA mRNA degradation system, we considered three new circuits where the yEGFP expression cassette TU1 or TU2 (modified with the addition of a pre-crRNA sequence) was integrated only once in the yeast genome. In contrast to (39), each ‘single-integration’ circuit showed a higher mRNA degradation (fluorescence inhibition) than the corresponding ‘double-integration’ variant. This was more evident when a single Rs was placed into the 5′ UTR. In particular, the strain containing a single integration of 5′ UTR-1xLb_Rs returned, under the action of dLb-NES, an 81.04-fold fluorescence inhibition, which improved the remarkable 60.46-fold reached by the corresponding double-integration version (see Figure 2E and Supplementary Figure S2B).

The results from the 5′ UTR engineering confirmed that the bare dFn cannot produce measurable effects on mRNA degradation. In contrast, dFn action becomes evident upon fusion to either two NLSs or NESs (as in the case of the 3′ UTR hosting pre-crRNAs). Each of the three versions of dFnCas12a showed a preference for the cognate pre-crRNA. Consistent with the trend detected on the 3′ UTR, the bare denAsCas12a displayed higher affinity than its two variants towards every pre-crRNA sequence placed in the 5′ UTR. Even though the mRNA degradation due to the three versions of dLbCas12a showed some difference between 3′ UTR and 5′ UTR, the corresponding fluorescence inhibition was always high.

Taken together, our results proved that dCasP—bare or fused to two copies of NLS or NES—degraded efficiently, in *S. cerevisiae*, mRNA strands containing pre-crRNA sequences into either the 5′- or 3′ UTR. A pre-crRNA hairpin in the 5′ UTR guaranteed higher degradation, which is consistent with (20). Moreover, the maximal fluorescence inhibition achieved by each of the three dCasP-NES on its own cognate repeat-spacer structure in the 5′ UTR (with one or two integrations) overcame 60-fold, i.e. it was higher than that reported by Csy4 in plants (up to 40-fold) (20). Furthermore, differently from the results in (39), our data show that mRNA degradation efficiency based on dCasP:pre-crRNA is not directly proportional to the expression level of the target mRNA.

To further prove the effectiveness of dCasP:pre-crRNA as a tool for mRNA degradation in *S. cerevisiae*, we applied it to a new target, namely the mRNA of the *Trametes trogii* Laccase 1 protein (Ttlcc1), which is an efficient biocatalyst of complex hydrocarbons. In a previous work from our lab, we expressed Ttlcc1 in *S. cerevisiae* and showed that, upon secretion, it degrades ABTS (2,2′-azinobis(3-ethyl-benzthiazoline-6-sulfonic acid)), which was supplied to SDC, conferring in this way a green-purple coloration to the growth medium (36). Here, we placed 1xFn_Rs upstream of the ATG of the *Ttlcc1* gene, i.e. in the 5′ UTR of pTEF2 (see Supplementary Figure S2C). We assessed the expression level of Ttlcc1—in the presence and absence of dFn-NES/denAs-NES—by exposing it to 1 mM CuSO_4_ and 0.5 mM ABTS in the SDC plates. In the absence of any dCasP, the plate color changed (see Figure 2F and Supplementary Figure S2D), indicating that Ttlcc1 had degraded ABTS after a 120-hour culture (plate no. 6). In contrast, the strains containing also a dCasP did not induce any relevant changes in the plate color. This was particularly evident upon expression of dFn-NES (plate no. 6 + pMM1450), whereas in the presence of denAs-NES (plate no. 6 + pMM1451) a pale reddish halo appeared around the cells. We concluded that most of the Ttlcc1 mRNA was degraded because of the action of dCasP-NES on 1xFn_Rs. In agreement with the results from FACS, dFn-NES appeared to have higher affinity than denAs-NES towards Fn_Rs, which determined a stronger mRNA degradation. Overall, these experiments pointed out that the dCas12a:pre-crRNA tool can be used to degrade, in *S. cerevisiae*, any mRNA modified with the insertion of a type V repeat-spacer structure.

### mRNA degradation based on pre-crRNA cleavage activity of (d)CasP is a general feature of CRISPR type V system

Since most of type V Cas12 proteins have the capability to cleave the pre-crRNA, we decided to explore whether other, less popular, Cas12 proteins could induce mRNA degradation after inserting their cognate pre-crRNA into the untranslated regions. To this goal, we selected two more proteins: dMbCas12a and Cas12j (also called Casϕ). In our previous work (30), we pointed out that dMbCas12a:crRNA was unfunctional as a transcriptional factor (both activator and repressor) in *S. cerevisiae*. Here, we checked if dMbCas12a:pre-crRNA could work as an mRNA degradation tool.

Casϕ proteins, from *Biggiephage clade*, are small-sized Cas proteins (about 70 up to 80 kDa) that were characterized by Pausch *et al.* (40). Three Casϕs (denoted as Casϕ1, Casϕ2 and Casϕ3) exhibited pre-crRNA cleavage activity. Among them, we chose Casϕ2 for our analysis.

**Figure 3.**
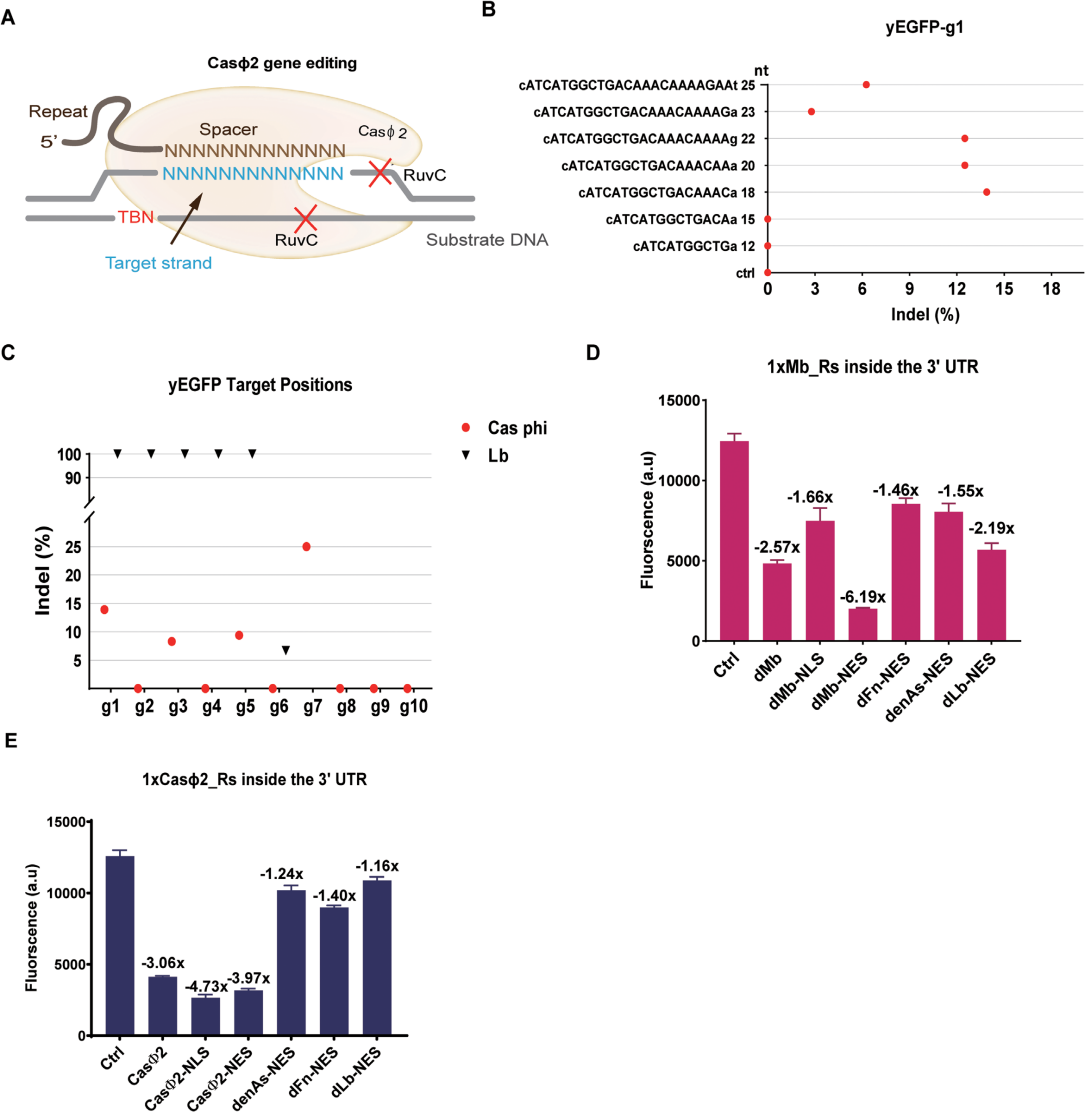
mRNA degradation, in *S. cerevisiae*, based on two more Cas proteins acting on their pre-crRNA placed into the mRNA 5′- or 3′ UTR. (**A**) The gene editing mechanism of Casϕ2. The complex Casϕ2:crRNA binds the target DNA sequence upon recognition of the PAM ‘TBN’, which enables the RuvC domain to carry out a double-strand break. (**B** and **C**) The genome editing efficiency of Casϕ2 in *S. cerevisiae*. (B) Editing efficiency as a function of crRNA length. ‘Ctrl’ (negative control) is a strain without crRNA expression such that Casϕ2 cannot bind and cleave the target DNA. (C) Editing efficiency at different target positions. Lb (i.e. LbCas12a) is our positive control. Compared to LbCas12a:crRNA, the genome editing due to Casϕ2:crRNA is rather inefficient. (**D** and **E**) mRNA degradation caused by dCasP and their variants. Both dMb (D) and Casϕ2 (E) lead to significant yEGFP reduction which means they can cleave their pre-crRNA in *S. cerevisiae* cells. Columns represent fluorescence mean values from at least three independent experiments (i.e. carried out in different days).

We first studied the genome editing efficiency of Casϕ2 in *S. cerevisiae* (we used a yeast codon-optimized Casϕ2 version) that had never been reported previously. As depicted in Figure 3A, the complex Casϕ2:crRNA binds the substrate DNA and induces a double-strand break upon recognition of the PAM ‘TBN’ (where B means not A). Initially we fixed, as a target, the g1 site, 452 bp downstream of the START codon of the *yEGFP* gene (see Supplementary Figure S3A) and tested the effect of crRNAs of different lengths on gene editing (see Figure 3B). The efficiency, in *S. cerevisiae*, of Casϕ2-driven genome editing turned out to be rather low, approaching only 15% when the crRNA length was equal to 18 nt. crRNAs shorter than 18 nt failed to induce DNA cleavage, whereas crRNAs longer than 22 nt were extremely inefficient (23 nt: 3%; 25 nt: 6%). We selected nine more target sites to see if genome editing could be more effective at different DNA locations. However, as shown in Figure 3C, only position g7 resulted cleaved slightly more effectively (25%) by Casϕ2, whereas LbCas12a reached 100% in five out of the six target sites. Hence, we had to conclude that Casϕ2 is not an optimal choice to carry out gene editing in *S. cerevisiae*.

To test dMb and Casϕ2 effects on mRNA degradation, we placed their cognate pre-crRNA sequences along the 3′ UTR following the *yEGFP* gene (see in Supplementary Figure S3B). Besides, we measured also the fluorescence repression in the presence of their variants containing either two NLSs or two NESs (dMb/Casϕ2–NLS and dMb/Casϕ2-NES). As depicted in Figure 3D, E, both dMb and Casϕ2 led to a significant reduction of yEGFP expression (dMb: 2.57-fold; Casϕ2: 3.06-fold), i.e. they provoked mRNA degradation by cleaving their pre-crRNA. dMb mRNA degradation activity was improved (6.19-fold) upon fusion to the two NES tags (dMb-NES) only. As for Casϕ2, both NLS and NES tag enhanced fluorescence inhibition: Casϕ2–NES reached 4.73-fold and Casϕ2–NLS 3.97-fold. None of the three dCas12a–NES used in the previous experiments appeared to work particularly well on the pre-crRNA of both MbCas12a and Casϕ2.

Type VI CRISPR-Cas systems target RNA molecules only. Thus, type VI Cas13 proteins have a double endoribonuclease activity since they can process their own pre-crRNA and cleave the target RNA sequence. Therefore, they might trigger the degradation of mRNA modified with the addition of their pre-crRNA sequences. However, a previous work from our lab showed that two commonly used Cas13 proteins (LwaCas13a and RfxCas13d) are unfunctional in *S. cerevisiae*, i.e. they cannot induce mRNA degradation upon making a complex with a properly designed crRNA (41). Here, we selected LwaCas13a (from *Leptotrichia wadei* (42)) to test if it could provoke the degradation of mRNA modified with the insertion of its pre-crRNA into the 5′ UTR upstream of the *yEGFP* gene. Beside LwaCas13a (yeast codon-optimized) we tested also LwaCas13a–NLS and LwaCas13a-NES. None of them reduced yEGFP expression significantly (see Supplementary Figure S3C) confirming our previous results (41).

On the whole, two more type V proteins, dMbCas12a and Casϕ2—and their variants containing either NLS or NES tags—can be used in *S. cerevisiae* as mRNA degradation tool even though they showed limitations in their applicability in yeast (dMbCas12a failed to act as transcriptional factors and Casϕ2 manifested low efficiency in genome editing). In contrast, type VI protein LwaCas13a resulted, again, unfunctional in *S. cerevisiae*. Thus, we think that a general property of only type V Cas proteins is the ability to induce the degradation of mRNA molecules containing their cognate pre-crRNA sequences.

### Chemical-mediated mRNA degradation

**Figure 4.**
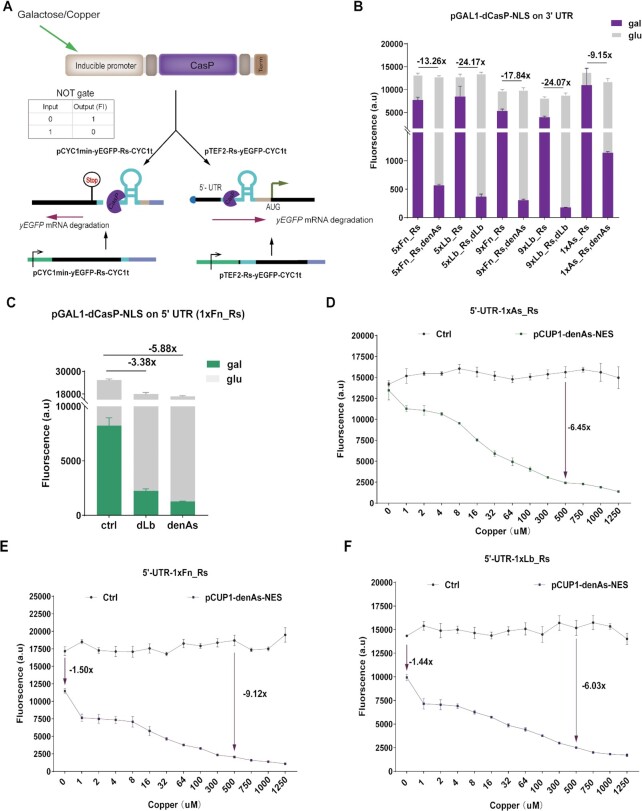
NOT gates based on mRNA degradation due to pre-crRNA cleavage by dCasP. (**A**) NOT gate mechanism. dCasP is produced under an inducible promoter, pGAL1 or pCUP1. Galactose or copper, as the system input, promotes the synthesis of dCasP (or its variants). In the absence of the input, the production of dCasP is hindered, which determines high yEGFP expression. In contrast, in the presence of the input, dCasP is synthesized and binds the mRNA encoding for yEGFP at the pre-crRNA and cut it nearby. As a result, mRNA degradation takes place and fluorescence decreases. (**B** and **C**) Galactose-inducible NOT gates. Values on top of adjacent columns represent the reduction (fold number) in fluorescence expression between the full and the control circuit (where dCasP–NLS is not expressed). In (B), we tested three kinds of pre-crRNA (Fn_Rs, Lb_Rs, and As_Rs) along the 3′ UTR downstream of the *yEGFP* gene, with a variable number of Rs (1, 5 or 9). Strains where denAs–NLS acted on Fn_Rs pre-crRNA sequences displayed higher fluorescence reduction by increasing the number of repeat-spacers (5xFn_Rs: 13.26-fold; 9xFn_Rs: 17.84-fold). This trend was, however, absent in the strains where dLb-NLS cleaved Lb_Rs pre-crRNAs (5xLb_Rs: 24.17-fold; 9xLb_Rs: 24.07-fold). A single As_Rs under the action of denAs-NLS returned the lowest fluorescence reduction (9.15-fold). (C) 1xFn_Rs pre-crRNA was inserted in the 5′ UTR upstream of the *yEGFP* gene. ‘Ctrl’ represents a strain in which dCasP expression cassette is missing. The two NOT gates based on this design appeared less effective than those hosting the pre-crRNA on the 3′ UTR of the same mRNA. (**D**) Strain 1, (**E**) strain 2.1 and (**F**) strain 3.1 response to increasing copper concentrations. mRNA degradation efficiency—and the consequent fluorescence inhibition—is positively correlated with the concentration of copper, when pCUP1 leads the expression of dAsCas12a–NES (the pre-crRNA was added to the 5′ UTR of the target mRNA). ‘Ctrl’ are strains lacking the expression cassette for dAsCas12a-NES. The values near the arrows indicate the fold of fluorescence reduction with respect to the control strains. Fluorescence levels are the mean values from at least three independent experiments, i.e. carried out in different days.

To create a chemical-inducible mRNA degradation system based on dCasP:pre-crRNA, we constructed, initially, NOT gates by expressing dCasP–NLS under the galactose-inducible *GAL1* promoter (see Figure 4A) instead of pGPD, as in the previous constructs. First, we placed dCasP pre-crRNA in the 3′ UTR downstream of the *yEGFP* gene. The results, presented in Figure 4B, show a significant reduction of fluorescence, in the presence of galactose, in every NOT gate. Four out of five strains displayed higher repression of fluorescence when utilizing pGAL1 instead of pGPD (see Supplementary Table S1). In particular, fluorescence inhibition due to the action of dLb–NLS on 5xLb_Rs and 9xLb_Rs increased from 17.66- to 24.17-fold and from 7.25- to 24.07-fold, respectively.

Next, we built two more galactose-sensitive NOT gates by placing the pre-crRNA sequence 1xFn_Rs along the 5′ UTR preceding the *yEGFP* gene. As depicted in Figure 4C, both NOT gates returned very different fluorescence levels in the presence or absence of galactose. Moreover, the strain expressing dLb-NLS displayed a slightly higher fluorescence repression than its counterpart where pGPD was used (3.38-fold versus 2.69-fold). In contrast, the strain hosting denAs–NLS performed less efficiently than the analogous one employing pGPD (5.88-fold versus 11.49-fold, see Supplementary Table S1). However, it should be noted that—as illustrated in Figure 4B, C—also the control strains, where the dCasP expression cassette is missing, showed a non-negligible reduction in fluorescence expression when growing in galactose instead of glucose. This is a consequence of the fact that yeast cells grow more slowly in galactose-containing media (see also (30)). To avoid this problem, we switched to the copper-inducible *CUP1* promoter (pCUP1) (43) to express dCasP–NES in the other NOT gates that we built in this work.

We chose three strains containing a different Rs on the 5′ UTR of the target mRNA (strain 1: As_Rs, strain 2: Fn_Rs, strain 3: Lb_Rs). Then, we integrated into each strain the plasmid expressing the cognate dCasP–NES under pCUP1. As shown in Figure 4D (denAs) and Supplementary Figure S4A, B (dLb and dFn), fluorescence decreased steadily, in the three strains, by increasing the concentration of copper sulfate, whereas it was stable in the control strain, where dCasP-NES was absent. Even though the highest mRNA degradation was reached at 1.25 mM copper, we found that this concentration induced a slight delay in yeast growth (compared to 500 μM copper—see Supplementary Figure S4C-E). Hence, we chose 500 μM copper as the best concentration to switch ON the *CUP1* promoter (‘0’ state of yEGFP expression). As a drawback, however, a significant promoter leakage was detected in strains 2 and 3, i.e. there was a non-negligible fluorescence decrease in the absence of copper (see Supplementary Figure S4A, B). We reasoned that the cause of this high fluorescence drop-off was the high affinity between dFn/dLbCas12a–NES and their cognate direct repeats (as reported in Figure 2D, both proteins induce a strong mRNA degradation in the presence of their cognate pre-crRNAs, which results in over 60-fold fluorescence reduction). Thus, we engineered two new versions of strains 2 and 3 (termed 2.1. and 3.1) by replacing dFn–NES and dLb–NES with denAs-NES. As expected, in both new strains the effects of promoter leakage on fluorescence expression were less evident (see Figure 4E-F). mRNA degradation at 500 μM copper was similar among strain 1 (6.45-fold fluorescence reduction), 2.1 (9.12-fold) and 3.1 (6.03-fold), although much lower than in strain 2 (29.29-fold) and 3 (44.05-fold—see Supplementary Figure S4A, B).

To sum up, we engineered galactose/copper-mediated mRNA degradation systems based on the nuclease activity of dCasP on pre-crRNA sequences. These constructs perform the NOT logic operation and can work as biosensors. Copper biosensors are more flexible than galactose ones since their detection range is rather wide (0–500 μM), and the output signal (fluorescence) shows significant changes already at the copper concentration of only 1 μM.

### AcrVA4 slightly recovered mRNA degradation caused by dLb-NLS

In our previous publication, we proved that three yeast codon-optimized type V anti-CRISPR proteins (AcrVA1, AcrVA4 and AcrVA5) considerably limit dCas12a functionality as a transcription factor in *S. cerevisiae* (30). Therefore, we checked if AcrVAs could also prevent mRNA degradation from the dCasP:pre-crRNA system. To this aim, we chose two strains: strain I containing 5xFn_Rs and denAs-NLS, and strain II hosting 5xLb_Rs and dLb-NLS. We integrated AcrVA1 (controlled by *GAL1* promoter) into both strains, whereas AcrVA4 and AcrVA5 were expressed (AcrVA4: pGPD, AcrVA5: pTEF1) in strains II only since they cannot act on AsCas12a (44–46). Inside strain II, only AcrVA4 recovered yEGFP expression level significantly in statistical terms, even though the increase in fluorescence was rather weak (2.14-fold, see Supplementary Figure S5A). AcrVA1 failed, in both strains, to stop mRNA degradation caused by dCasP-NLS (see Supplementary Figure S5B). Our results ensure that AcrVA4 hinders, though only partially, mRNA degradation by dCasP:pre-crRNA. Probably, fluorescence recovery can be improved by making adjustments on the whole circuit, such as using less repeat-spacers on the target mRNA and expressing a bare dCasP.

### Construction of two-input boolean gates based on mRNA degradation

In 2019, Sato *et al.* engineered a new dLbCas12a-based activator by splitting dLbCas12a into N and C parts. This solution outperformed previous activators, based on the full dSpCas9 and dLbCas12a, when targeting endogenous genes in human cells (28). Furthermore, Qi *et al.* accomplished the construction of multiple-input/output logic circuits, in mammalian cells, by means of the split dLbCas12a system (31).

**Figure 5.**
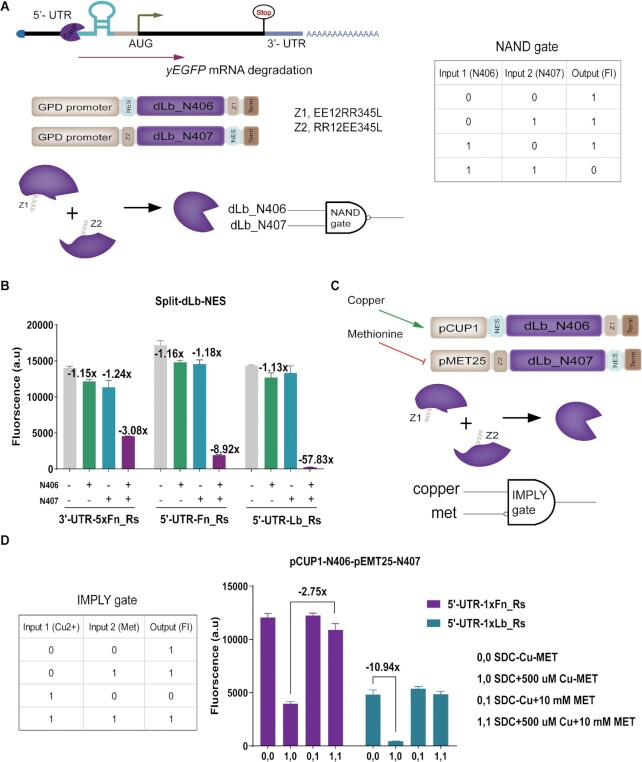
NAND and IMPLY gates based on split dLb–NES and mRNA degradation. (**A**) NAND gate diagram. The two halves of dLb–NES (fused to leucine zippers) are placed downstream of the strong *GPD* promoter. Upon expression, the two halves reconstitute a complete dLb–NES that triggers mRNA degradation by cleaving a pre-crRNA sequence. (**B**) NAND gate performance. yEGFP expression displayed a significant decrease in the presence of both halves of dLb-NES. Values above the columns represent the fold of fluorescence reduction with respect to ‘0,0’ strain, where both N406 and N407 are not expressed. (**C**) Scheme of an IMPLY gate sensing copper and methionine. Differently from (A), both N406 and N407 lie downstream of a promoter controlled by a chemical. (**D**) IMPLY gate performance. The highest reduction in yEGFP expression is achieved with copper (500 μM) and no methionine (‘1,0’ state). Fluorescence levels are the mean values from at least three independent experiments, i.e. carried out in different days.

Moving from these works (28,31), we exploited a split Cas protein to build, initially, a NAND gate based on the dCasP:pre-crRNA system. As depicted in Figure 5A, we separated dLb–NES into two parts termed N406 and N407 (the former comprised the first 406 amino acids of dLb-NES, the latter started from the 407^th^ amino acid). Both N406 and N407 were produced by the strong constitutive *GPD* promoter. In order to enhance the probability of interaction between N406 and N407, we fused them to the leucine zipper domains Z1 (N406) and Z2 (N407) (31). We realized three versions of a NAND gate by employing each time a different pre-crRNA. As illustrated in Figure 5B, every NAND gate returned a dramatic repression of yEGFP expression only in the presence of the two halves of dLb-NES. In contrast, either N406 or N407 alone could not trigger efficient mRNA degradation. The strain hosting dLbCas12a cognate pre-crRNA reached a remarkable 57.83-fold fluorescence reduction with respect to the control strain where both halves of dLb–NES were missing. This result is equivalent to the 96% of the fluorescence inhibition reached by the full dLb-NES—see Supplementary Figure S6A.

This NAND gate did not respond to any chemical since the two inputs were the dLb–NES halves. Thus, we placed the expression of N406 and N407 under the control of two small molecules (copper and galactose) by replacing pGPD with pCUP1 and pGAL1, respectively—see Supplementary Figure S6B. Therefore, yEGFP expression should be switched OFF only in the presence of both inducers. However, the gate did not behave as expected and returned a too low fluorescence signal in the presence of the only galactose. This outcome can be imputed to pCUP1 high leakage (see Supplementary Figure S6C).

To build a Boolean gate properly responding to two input chemicals, we replaced pGAL1 with pMET25 (47) whose transcriptional activity is inhibited in the presence of methionine. In this way, we realized an IMPLY gate responsive to copper and methionine (see Figure 5C). First, we constructed a methionine-responsive YES gate where *denAs-NES* was placed downstream of pMET25 and targeted 1xFn_Rs on the 5′ UTR of the reporter mRNA (see Supplementary Figure S6D). This circuit permitted us to assess that 10 mM methionine inhibited pMET25 activity up to around 90 % (see Supplementary Figure S6D and Table S2). Hence, this concentration would be adopted also with the IMPLY gate.

We realized two versions of an IMPLY gate by employing, as a target, 1xFn_Rs and 1xLb_Rs on the 5′UTR preceding the yEGFP. As expected, both IMPLY gates showed a drastic decrease in fluorescence expression when exposed to the sole copper (see Figure 5D and Supplementary Figure S6E) reaching an ON/OFF ratio equal to 10.94 when 1xLb_Rs was targeted. The relatively low fluorescence levels associated with the ‘1’ outputs are probably due to both pCUP1 leakage and the strong affinity between dLb-NES and its cognate repeat sequence. However, the opposite actions of copper and methionine on their corresponding promoters cancelled out any possible negative effects of the promoters’ leakage on the logic behavior of the whole circuit.

Overall, our circuits showed that two-input logic gates can be built by using a split dCas protein together with the dCasP:pre-crRNA system for protein degradation. In order to reduce the number of TUs in our NAND and IMPLY gates, we recurred to inducible promoters. However, pCUP1 leakage prevented a correct reproduction of the NAND gate truth table. A working, fully inducible, NAND gate requires to replace pCUP1 either with a different, less leaky, inducible promoter or a more complex scheme to control transcription, such as a one-step cascade (see Supplementary Figure S7).

## CONCLUSIONS

We have developed a new efficient tool for mRNA degradation, in *S. cerevisiae*, based on type V CRISPR-Cas systems. The method requires to express a dCas12a protein and insert a (cognate or non-cognate) pre-crRNA sequence into the 5′- or 3′ UTR of the target mRNA (here *yEGFP* or *Ttlcc1*). We tested three commonly used type V (d)Cas12a proteins i.e. dFnCas12a, (d)LbCas12a denAsCas12a and their variants (dCasP–NLS and dCasP-NES). We found that the bare dFnCas12a cannot degrade mRNA well unless fused to the NLS or NES tag. Moreover, it displays a clear preference for the cognate pre-crRNA. In contrast, the bare denAsCas12a outperforms its two variants, i.e. denAs-NLS and denAs-NES. Even though the behavior of dLbCas12a and its two variants did non highlight a precise pattern, they appear to always inhibit yEGFP expression strongly. Besides, consistent with (20), mRNA degradation is higher when the pre-crRNA is placed along the 5′ UTR. Indeed, the strongest mRNA degradation we achieved caused a 81.04-fold fluorescence reduction with the pre-crRNA on the 5′ UTR and a 18.59-fold fluorescence inhibition with the pre-crRNA on the 3′ UTR. Moreover, mRNA degradation efficiency based on dCasP:pre-crRNA does not depend on the target mRNA concentration. We also pointed out that mRNA degradation based on the endoribonuclease activity of CRISPR-Cas might be a general property of type V Cas proteins since two more proteins of this family, i.e. dMbCas12a and Casϕ2 did repress yEGFP expression, whereas type VI LwaCas13a was unfunctional in *S. cerevisiae* by targeting its repeat-spacer structure on the mRNA. Following these results, we applied our new mRNA degradation tool to the construction of Boolean gates. We realized NOT gates sensing galactose or copper; YES gates responsive to methionine; and IMPLY gates regulated by copper and methionine. The latter made use of a split dLbCas12a-2xNES system.

On the whole, the dCasP:pre-crRNA degradation tool established in this work is a flexible and easy-to-implement solution to control gene expression at the mRNA level. The importance of modulating translation, beside transcription, to build complex gene networks has been known since over a decade (48). In *S. cerevisiae*, important progress has been made with the re-engineering of the RNAi pathway (49) and its later optimization (50,51). As a drawback, this approach puts a non-negligible metabolic burden on yeast cells since it requires to express two foreign proteins (the Argonaute and the Dicer) together with a long double-stranded RNA molecule, i.e. the source of the small-interfering RNAs (siRNAs). Direct control of translation, via chemicals, has been made possible through the usage of riboswitches/ribozymes that, like the pre-crRNA sequences in our tool, are generally placed on the 5′ UTR of the target genes (52,53). A handful of bacterial riboswitches/ribozymes have been proved to be functional in *S. cerevisiae*, whereas engineering new ones is a difficult task that requires to compute the exact secondary structures in which the riboswitches/ribozymes shall fold to first bind chemicals and then exert the desired action on the mRNA (7). Repression of translational can be also achieved through artificial PUF proteins. They are assembled *de novo* in a modular fashion (54) that reminds of the TAL-Effectors (55). However, PUF proteins present severe limitations in the kind and length of sequences they can recognize and bind along the mRNA, which has hindered, so far, their use in synthetic gene circuits (56). It should be noted, finally, that a recent work by Otoupal *et al.* (57) has shown how to transform a type VI Cas13 protein into a ribosome-recruiting unit i.e. an activator of translation. Although implemented in *E. coli*, which poses less troubles than eukaryotic cells, this last chimeric protein shows the significance and the attention that is currently paid to the engineering of novel Synthetic Biology tools for translation regulation.

**Figure 6.**
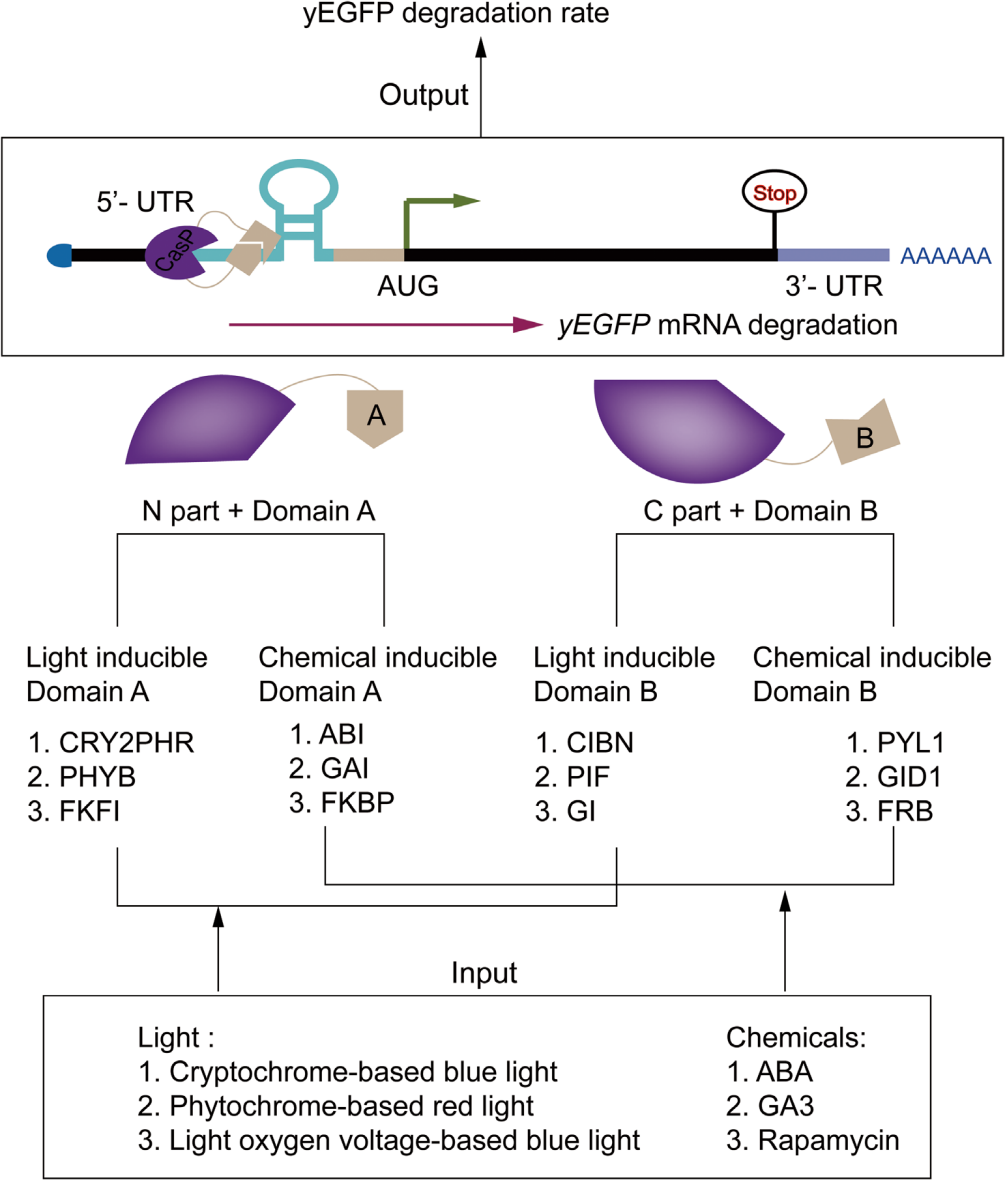
Novel biosensors design based on dCasP:pre-crRNA and the inducible split dLbCas12a. The two halves of the split dLbCas12a are fused to light/chemical-inducible dimerization domains (58) such that they reconstitute a complete dLbCas12a only in the presence of an external light or chemical signal. dLbCas12a, then, cleaves the pre-crRNA sequence on the 5′ UTR of the mRNA encoding for the reporter protein (yEGFP). Hence, a decrease in fluorescence highlights the presence of a particular substance (or light source) in the environment.

The dCasP:pre-crRNA mRNA degradation tool demands to modify the target mRNA with functional hairpin structures that, differently from riboswitches/ribozymes, do not demand any further engineering. Moreover, it works by using type V (d)Cas proteins that can be expressed in highly amount in yeast cells without inducing toxicity (30). Overall, dCasP:pre-crRNA provides an easy solution to repress translation by inducing mRNA degradation. We have shown that it can be applied to the building of gene Boolean gates straightforwardly. Thus, it could allow the construction of novel biosensors (see Figure 6) or even larger digital circuits for DNA computing.

## DATA AVAILABILITY

FCS files can be obtained from:

http://flowrepository.org/id/RvFr5Rf84Cyc2goLJIaKRKPyovzjpnwlKRpznnHPZ2N wBbqkSpfUzdXtO1dx7wdZ and http://flowrepository.org/id/RvFrTgEVxRx5ukH1eDBqOv77q9WZkbcTpcP39mXC1tO5CIBtCiIMZfu27xDb0SIc.

## Supplementary Material

gkac1270_Supplemental_Files
