# Supplementary material for: CRISPR-associated type V proteins as a tool for controlling mRNA stability in S. cerevisiae synthetic gene circuits
Source: Nucleic Acids Res. 2023 Jan 18;51(3):1473–87. doi: 10.1093/nar/gkac1270 (PMC9943656; doi:10.1093/nar/gkac1270)
Supplement: gkac1270_Supplemental_Files [file gkac1270_supplemental_files.zip › LY-MAM-SupplementaryMaterial-rev.pdf]

Supplementary material

CRISPR-associated type V proteins as a tool for for controlling mRNA stability in *S. cerevisiae* synthetic gene circuits

Lifang Yu[1+], and Mario Andrea Marchisio[1*]

[1] School of Pharmaceutical Science and Technology, Tianjin University, 92 Weijin Road, 300072-Tianjin, China

Results

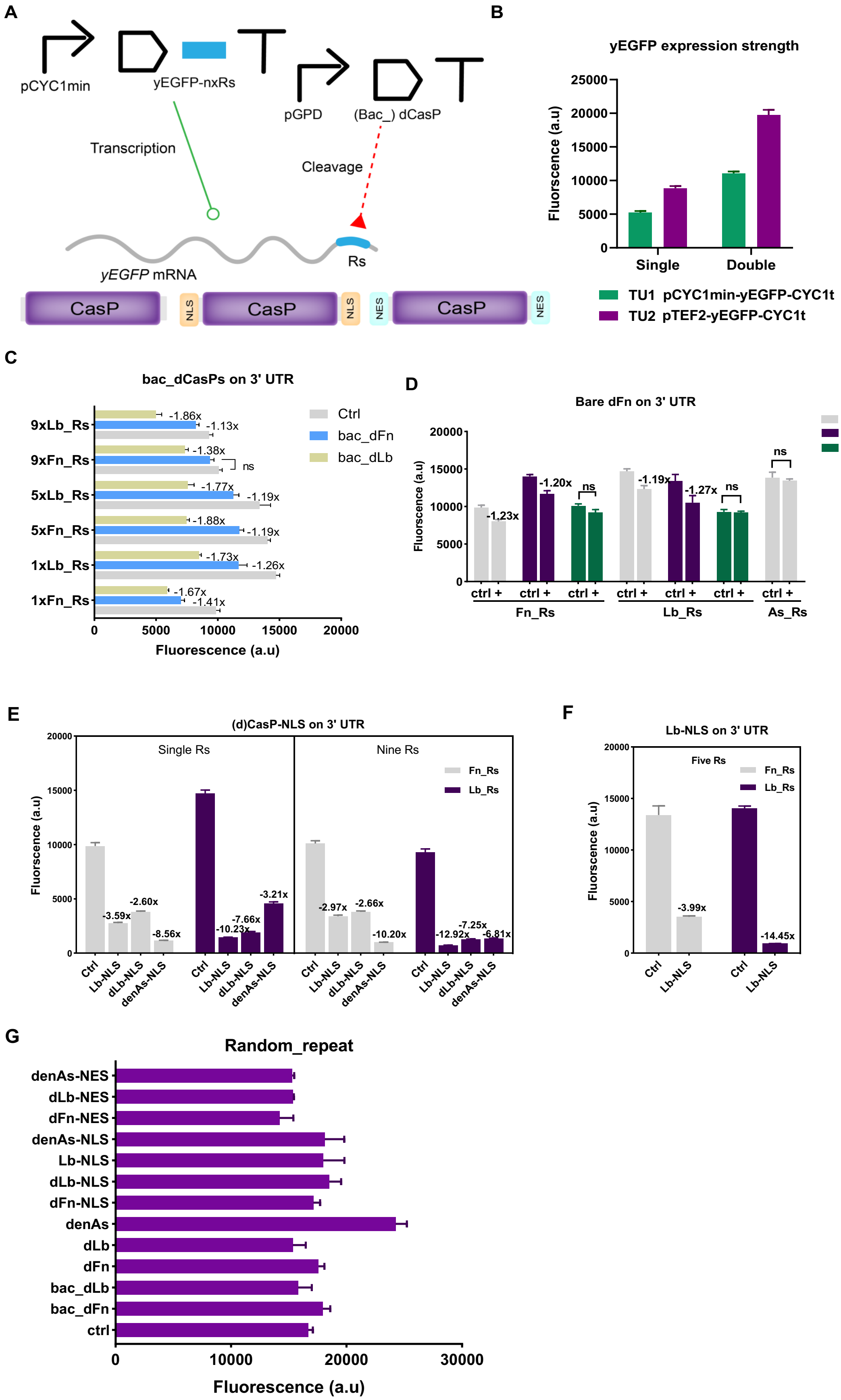
A
pCYC1min
yEGFP-nxRs
pGPD
(Bac_) dCasP
Transcription
Cleavage
yEGFP mRNA
Rs
CasP
NLS
CasP
NLS
NES
CasP
NES
B
yEGFP expression strength
Fluorscence (a.u)
25000
20000
15000
10000
5000
0
Single
Double
TU1 pCYC1min-yEGFP-CYC1t
TU2 pTEF2-yEGFP-CYC1t
C
bac_dCasPs on 3' UTR
9xLb_Rs
9xFn_Rs
5xLb_Rs
5xFn_Rs
1xLb_Rs
1xFn_Rs
-1.86x
-1.13x
-1.38x
ns
-1.77x
-1.19x
-1.88x
-1.19x
-1.73x
-1.26x
-1.67x
-1.41x
Ctrl
bac_dFn
bac_dLb
0
5000
10000
15000
20000
Fluorescence (a.u)
D
Bare dFn on 3' UTR
1x
5x
9x
Fluorscence (a.u)
20000
15000
10000
5000
0
-1.23x
-1.20x
ns
-1.19x
-1.27x
ns
ns
ctrl +
Fn_Rs
Lb_Rs
As_Rs
E
(d)CasP-NLS on 3' UTR
Single Rs
Nine Rs
Fn_Rs
Lb_Rs
Fluorescence (a.u)
20000
15000
10000
5000
0
-3.59x
-2.60x
-8.56x
-10.23x
-7.66x
-3.21x
-2.97x
-2.66x
-10.20x
-12.92x
-7.25x
-6.81x
Ctrl
Lb-NLS
dLb-NLS
denAs-NLS
F
Lb-NLS on 3' UTR
Five Rs
Fn_Rs
Lb_Rs
Fluorscence (a.u)
-3.99x
-14.45x
Ctrl
Lb-NLS
G
Random_repeat
denAs-NES
dLb-NES
dFn-NES
denAs-NLS
Lb-NLS
dLb-NLS
dFn-NLS
denAs
dLb
dFn
bac_dLb
bac_dFn
ctrl
0
10000
20000
30000
Fluorescence (a.u)

Figure S1. mRNA degradation in *S. cerevisiae* based on dCasP-pre-crRNA interaction at the mRNA 3' UTR. (**A**) Circuit diagram. One transcription unit (TU) encodes for the yEGFP, whose gene is placed downstream of a *CYC1* minimal promoter (pCYC1min). Moreover, a CasP pre-crRNA sequence, made of *n=1, 5, or 9* repeat-spacer (Rs) motifs, is added after the STOP codon. The other TU expresses (bac_)dCasP via the strong constitutive *GPD* promoter (pGPD). The dCasP configurations used in this work are: bare dCasP, dCasP-NLS, and dCasP-NES. (**B**) yEGFP expression levels without pre-crRNAs on the mRNA. TU1 was adopted for the initial tests of dCasP:pre-crRNA efficiency, i.e., when the pre-crRNAs were placed on the mRNA 3' UTR. TU2 was used in later tests (see below) with the pre-crRNA inserted along the 5' UTR. "Single" and "double" refer to the number of integrations into the yeast genome of the plasmids carrying TU1 or TU2. In order to make the effects of mRNA degradation more evident, we used yeast strains with a double integration of TU1 modified with pre-crRNAs on the 3' UTR and TU2 altered with one Rs on the 5' UTR. (**C**) mRNA degradation effects due to bare bacterial dCasPs (bac_dCasPs) acting on pre-crRNA sequences in the 3' UTR. The highest yEGFP inhibition was achieved by bac_dLb on 5xFn_Rs (1.88-fold). (**D**) The yeast codon optimization of the bare dFnCas12a does not improve the results of bac_Fn in (C). (**E-F**) Effects of (d)CasP-NLS variants on mRNA degradation. 'Single Rs' or 'Nine Rs' refers to the pre-crRNA configuration. 'Ctrl' are strains that do not contain (d)CasP-NLS. (**G**) mRNA degradation is absent in strains containing random pre-crRNAs.

Values on the top of columns represent the ratio between the fluorescence intensity of the control and the full-circuit strain. Mean fluorescence values were calculated on at least three independent FACS experiments, i.e., carried out in different days. 'ns' means no statistically significant difference (p-value > 0.05; two-sided Welch's *t* test).

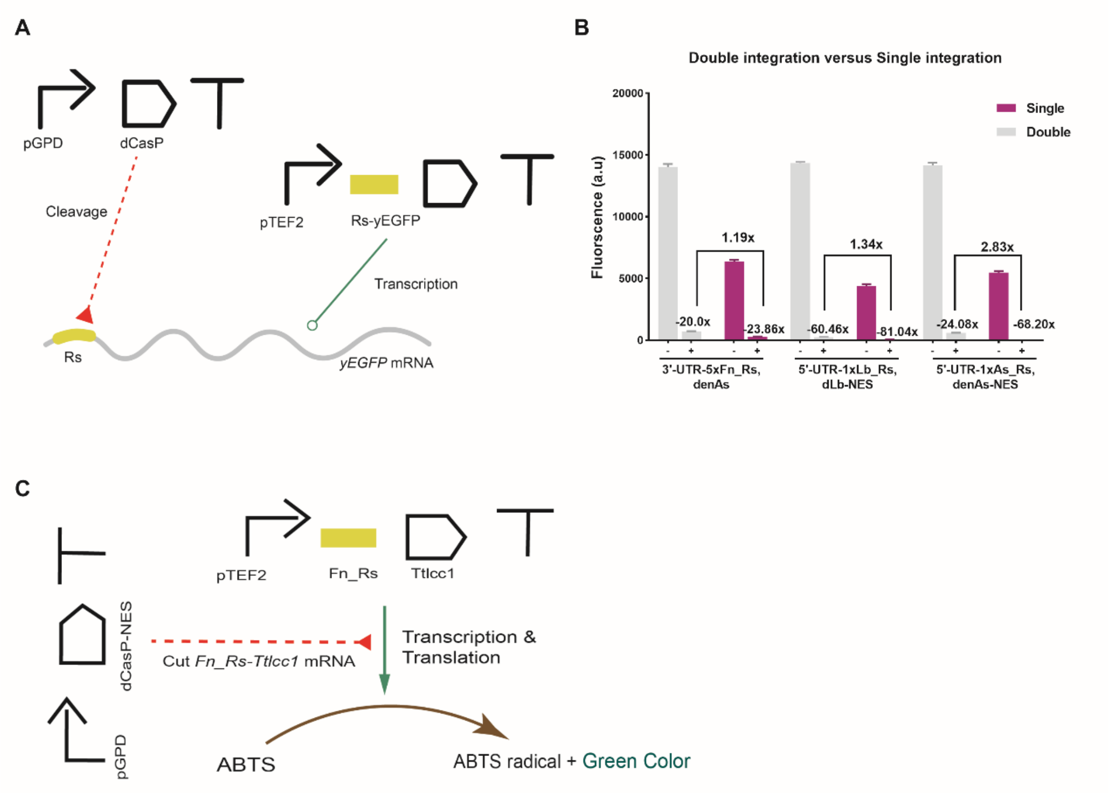
A
pGPD
dCasP
Cleavage
pTEF2
Rs-yEGFP
Transcription
Rs
yEGFP mRNA
B
Double integration versus Single integration
Single
Double
Fluorscence (a.u)
20000
15000
10000
5000
0
1.19x
1.34x
2.83x
-20.0x
-23.86x
-60.46x
-81.04x
-24.08x
-68.20x
3'-UTR-5xFn_Rs, denAs
5'-UTR-1xLb_Rs, dLb-NES
5'-UTR-1xAs_Rs, denAs-NES
C
pTEF2
Fn_Rs
Ttlcc1
dCasP-NES
pGPD
Cut Fn_Rs-Ttlcc1 mRNA
Transcription & Translation
ABTS
ABTS radical + Green Color

D

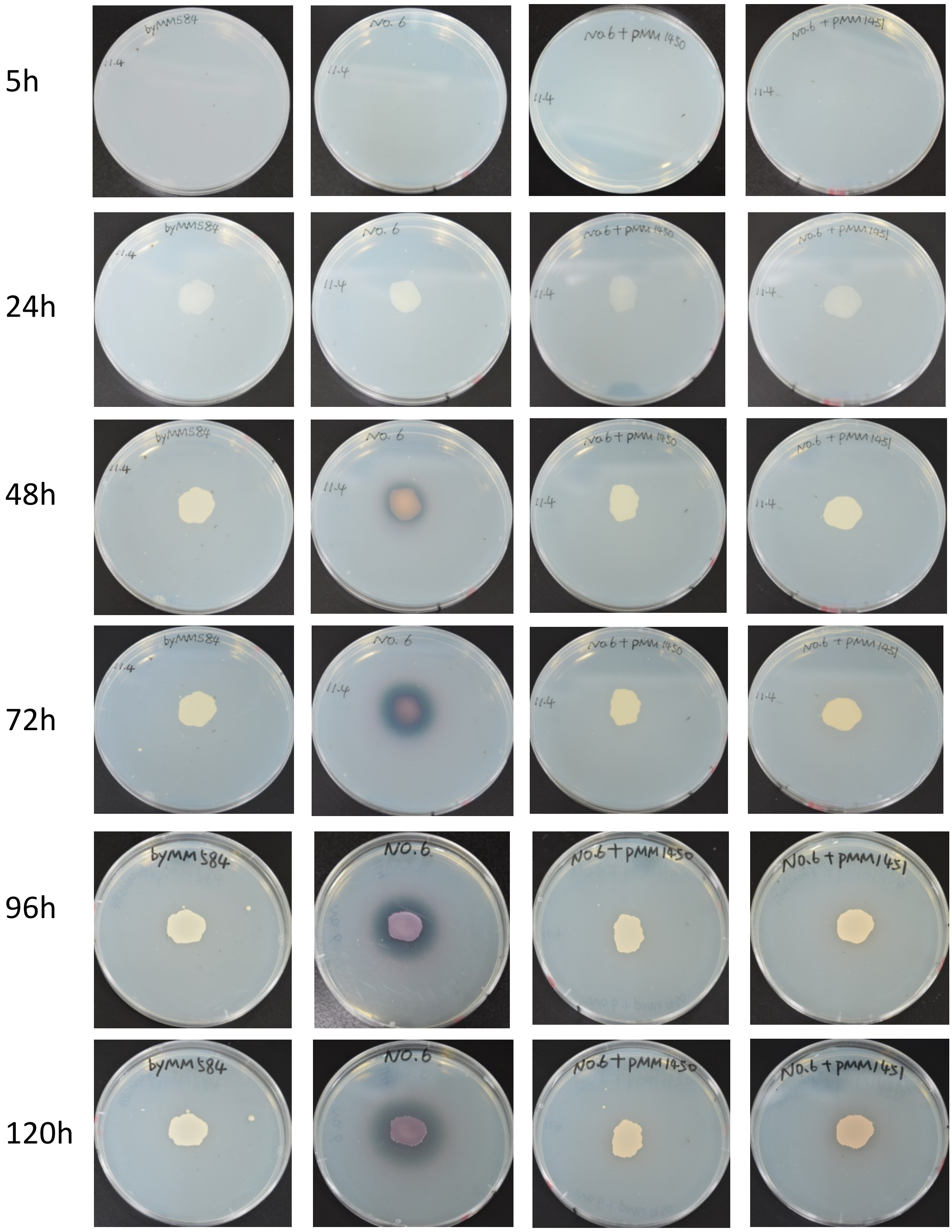

Figure S2. mRNA degradation based on the dCasP:pre-crRNA system with the pre-crRNA placed on the 5′ UTR. (**A**) Circuit diagram. Since a longer 5′ UTR might decrease the expression of the yEGFP, we selected strains with a double integration of pTEF2-Rs-yEGFP-CYC1t to finalize the circuit with the further integration of the dCasP-containing plasmid. dCasP was always placed downstream of the strong *GPD* promoter. (**B**) mRNA degradation efficiency: comparison between strains with double and single integration of yEGFP TU (double/single integration mean high/low level of *yEGFP* mRNA expression). The single-integration strains showed higher fluorescence reduction than the double-integration ones. Fluorescence levels are the mean values from at least three independent experiments, which were carried out in different days. (**C**) Circuit expressing Ttlcc1. The sequence 1xFn_Rs was placed upstream of the START codon of *Ttlcc1*. Once *Ttlcc1* is transcribed, dCasP-NES

(either dFn-NES or denAs-NES) recognizes Fn_Rs and cleaves it, decreasing the expression of Ttlcc1. As a results, the conversion of ABTS into ABTS radicals and a purple-green pigment is weakened. (**D**) Characterization of Ttlcc1 activity. Four strains were used, i.e., the background strain byMM584; the strain containing the sole Ttlcc1 expression cassette ("NO.6"); and two other strains, based on "NO.6", where either pMM1450 (dFn-NES) or pMM1451 (denAs-NES) was integrated. All strains were grown in SDC plates supplemented with 1 mM $CuSO_4$ and 0.5 mM ABTS. Pictures were taken every 12 hours.

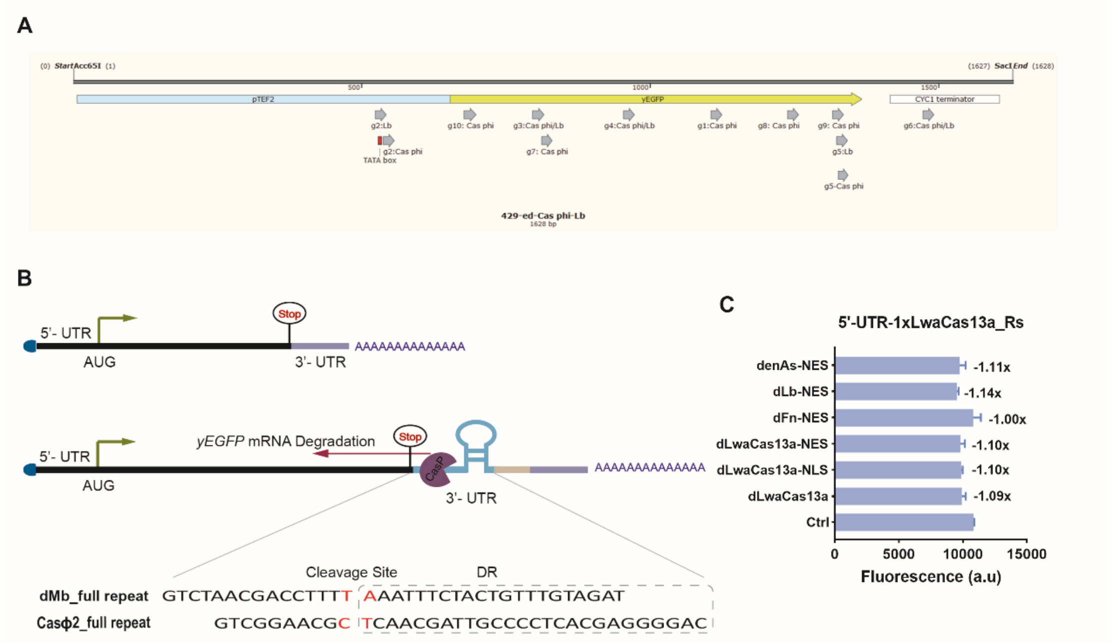

Figure S3. (**A**) Map of the target sites used to characterize Casϕ2-based genome editing. (**B**) The mRNA degradation mechanism based on dMb/Casϕ2:pre-crRNA (in the diagram, the pre-crRNA is inserted only in the 3' UTR of *yEGFP* gene that was controlled by the minimal *CYC1* promoter). The red bases represent the cleavage sites for dMb and Casϕ2. (**C**) mRNA degradation caused by type VI LwaCas13a. The reduction of fluorescence due to this CRISPR-associated protein is negligible. The mean value of each fluorescence level comes from at least three independent experiments that were carried out in different days.

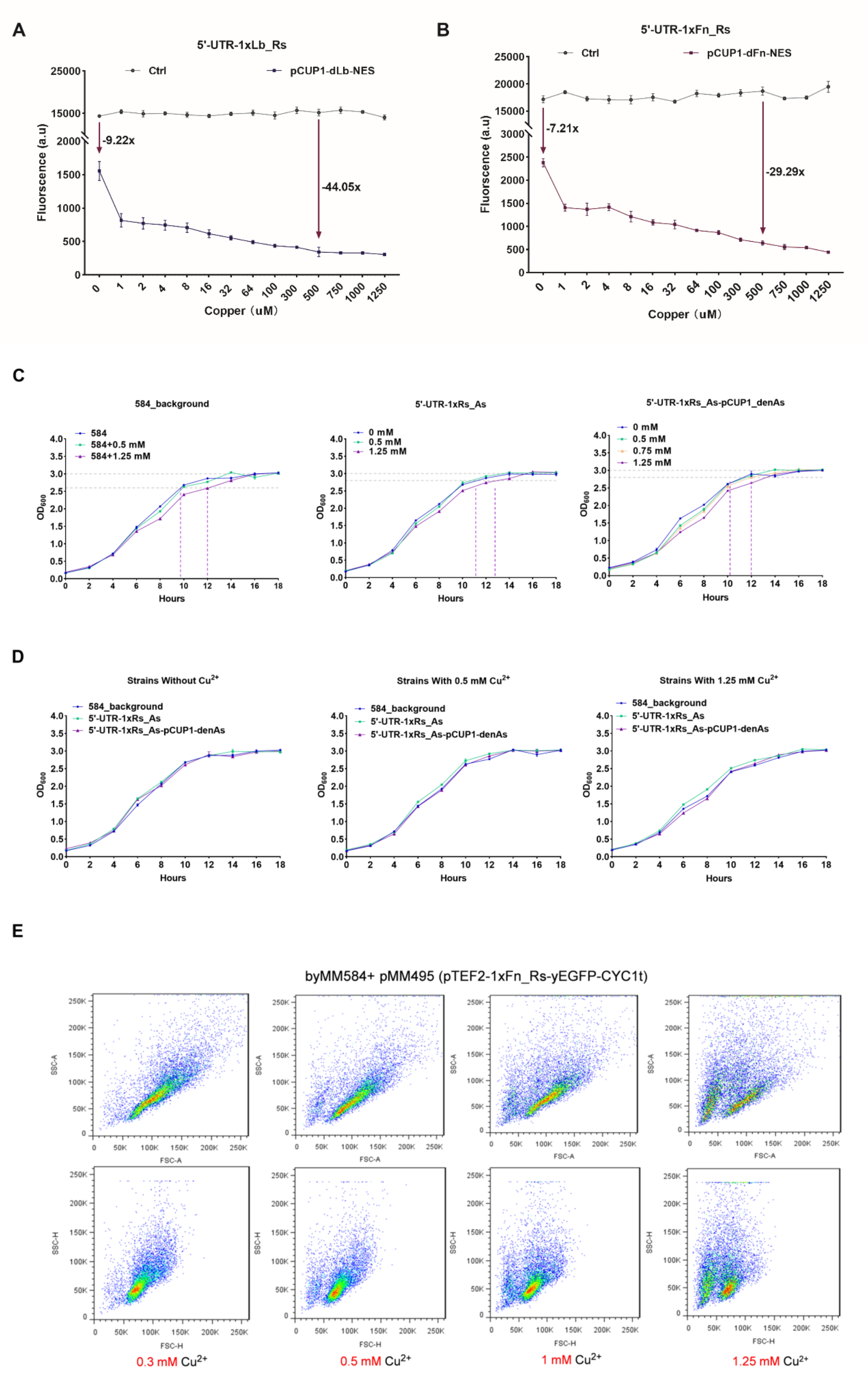

Figure S4. mRNA degradation regulated by galactose or copper. (**A**) Strain 2 and (**B**) strain 3

response to increasing copper concentrations. The values close to the arrows represent the fluorescence inhibition fold with respect to the control strains (i.e., without dCasP-NES expression). (**C**) and (**D**): growth curves. We analyzed three strains: byMM584 (our chassis), strain 1, and the corresponding control strain. (C) Each strain grows more slowly in the presence of 1.25 mM copper (especially if compared to 500 µM of copper). (D) The growth delay has nothing to do with the synthetic content of yeast cells and only depends on the concentration of copper. (**E**) The shape of a yeast cell population changes with increasing concentrations of copper.

Repression Folds			
Position	Name	pGPD	pGAL1
3'-UTR	5xFn_Rs-denAs-NLS	15.26	13.26
	5xLb_Rs-dLb-NLS	17.66	24.17
	9xFn_Rs-denAs-NLS	10.20	17.84
	9xLb_Rs-dLb-NLS	7.25	24.07
	1xAs_Rs-denAs-NLS	3.43	9.15
5'-UTR	1xFn_Rs-dLb-NLS	2.69	3.38
	1xFn_Rs-denAs-NLS	11.49	5.88

Table S1. The repression folds of yEGFP expression when dCasP is placed under pGPD or pGAL1. Only two out of seven strains, i.e., 5xFn_Rs-denAs-NLS and 1xFn_Rs-denAs-NLS, showed higher yEGFP repression under the pGPD rather than pGAL1.

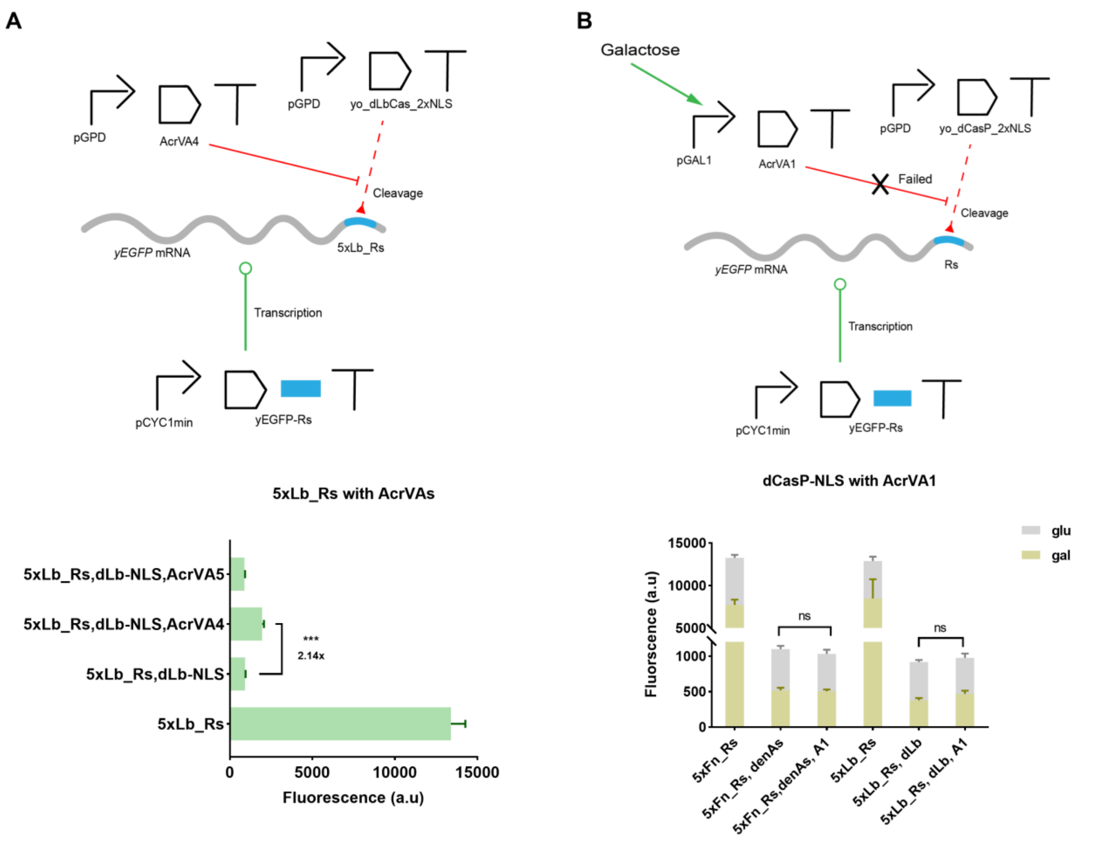

Figure S5. Anti-CRISPR action on dCasP:pre-crRNA. (**A**) Strains hosting AcrVA4 performed a slight recovery of the yEGFP expression level, which means that AcrVA4 spoils the affinity between dLb-NLS and its pre-crRNA—here 5xLb_Rs. (**B**) AcrVA1 expressed under the inducible *GAL1* promoter. Only in the presence of galactose, AcrVA1 is produced. However, AcrVA1 cannot prevent pre-crRNA cleavage by denAs-NLS and dLb-NLS, since no strains displayed any fluorescence recovery.
Mean fluorescence levels were calculated on at least three independent experiments, i.e., carried out in different days. 'ns' means no statistically significant difference (p-value > 0.05; two-sided Welch's *t* test). '***' indicates significant statistical difference (p-value < 0.001; two-sided Welch's *t* test).

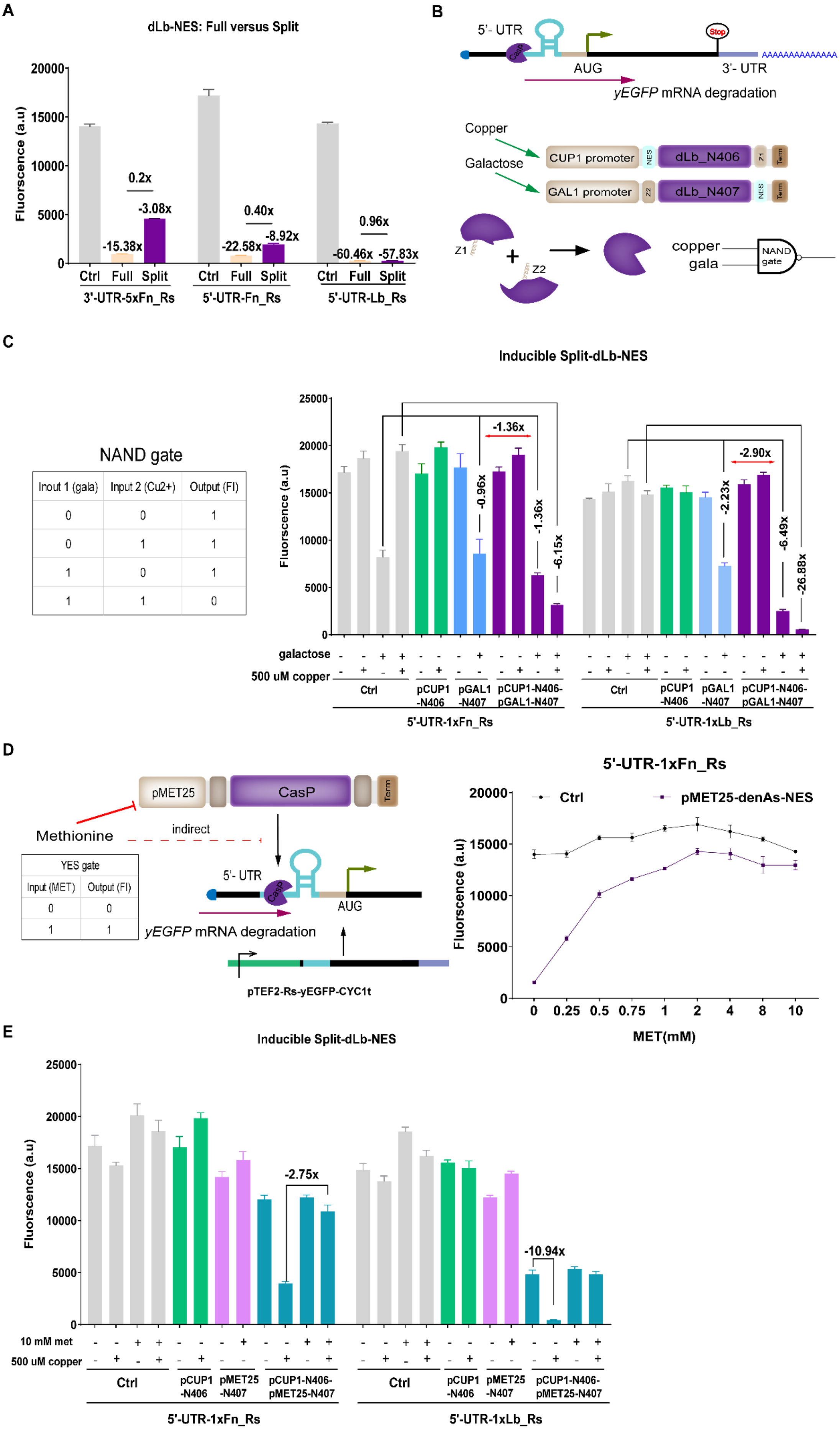
A
dLb-NES: Full versus Split
Fluorescence (a.u)
0.2x
-3.08x
-15.38x
0.40x
-8.92x
-22.58x
0.96x
-60.46x
-57.83x
Ctrl Full Split
3'-UTR-5xFn_Rs
5'-UTR-Fn_Rs
5'-UTR-Lb_Rs
B
5'- UTR
AUG
3'- UTR
yEGFP mRNA degradation
Copper
Galactose
CUP1 promoter
dLb_N406
GAL1 promoter
dLb_N407
NES
Term
Z1
Z2
copper
gala
NAND gate
C
NAND gate
Inout 1 (gala) | Input 2 (Cu2+) | Output (FI)
0 | 0 | 1
0 | 1 | 1
1 | 0 | 1
1 | 1 | 0
Inducible Split-dLb-NES
-1.36x
-0.96x
-1.36x
-6.15x
-2.90x
-2.23x
-6.49x
-26.88x
galactose
500 uM copper
Ctrl
pCUP1 -N406
pGAL1 -N407
pCUP1-N406- pGAL1-N407
5'-UTR-1xFn_Rs
5'-UTR-1xLb_Rs
D
pMET25
CasP
Term
Methionine
indirect
YES gate
Input (MET) | Output (FI)
0 | 0
1 | 1
5'- UTR
AUG
yEGFP mRNA degradation
pTEF2-Rs-yEGFP-CYC1t
5'-UTR-1xFn_Rs
Ctrl
pMET25-denAs-NES
Fluorescence (a.u)
MET(mM)
E
Inducible Split-dLb-NES
Fluorescence (a.u)
-2.75x
-10.94x
10 mM met
500 uM copper
Ctrl
pCUP1 -N406
pMET25 -N407
pCUP1-N406- pMET25-N407
5'-UTR-1xFn_Rs
5'-UTR-1xLb_Rs

Figure S6. (**A**) Comparison between the performance of split and full dLb-NES. When acting on a cognate pre-crRNA, the two configurations did not show an evident difference (57.83-fold and 60.46-fold fluorescence reduction, respectively). In contrast, in the presence of a noncognate pre-crRNA (3' UTR-5xFn_Rs and 5' UTR-Fn_Rs), the full dLb-NES appeared more effective. (**B**) NAND gate sensing copper and galactose. Differently from Figure 5A, both N406 and N407 lie downstream of an inducible promoter. Thus, the mRNA degradation is regulated by two chemicals. (**C**) NAND gate performance. The highest reduction in yEGFP expression demands both galactose (2%) and copper (500 µM). pCUP1 presents a non-negligible leakage (quantified in the values above the red arrows) that spoils the "1" output (too low) corresponding to "1" galactose and "0" copper. (**D**) YES gate based on the *MET25* inducible promoter. Methionine suppresses the transcriptional action of pMET25, which prevents the synthesis of denAs-NES. Hence, mRNA degradation is inhibited as well. The titration curve shows how the YES gate works at diverse concentrations of methionine, from 0 to 10 mM. (**E**) IMPLY gates sensing copper and methionine. yEGFP displayed a significant decrease when the complete circuit (see Figure 5C) is induced with 500 µM copper only.
Fluorescence levels are the mean values from at least three independent experiments, i.e., carried out in different days.

Concentration (mM)	mean ctrl	mean with denAs-NES	ratio (Ctrl/with denAs-NES)
0	14004.01	1538.5	9.10
0.25	14055.94	5805.67	2.42
0.5	15626.76	10168.86	1.54
0.75	15629.36	11611.33	1.35
1	16522.95	12631.43	1.31
2	16900.13	14268.91	1.18
4	16222.13	14072.59	1.15
8	15483.43	12966.23	1.19
10	14279.58	12961.22	1.10

Table S2. Values from the methionine titration curve in Figure S6D. When methionine concentration is bigger than or equal to 2 mM, the ratio between the fluorescence levels in the absence and the presence of denAs-NES is almost 1.

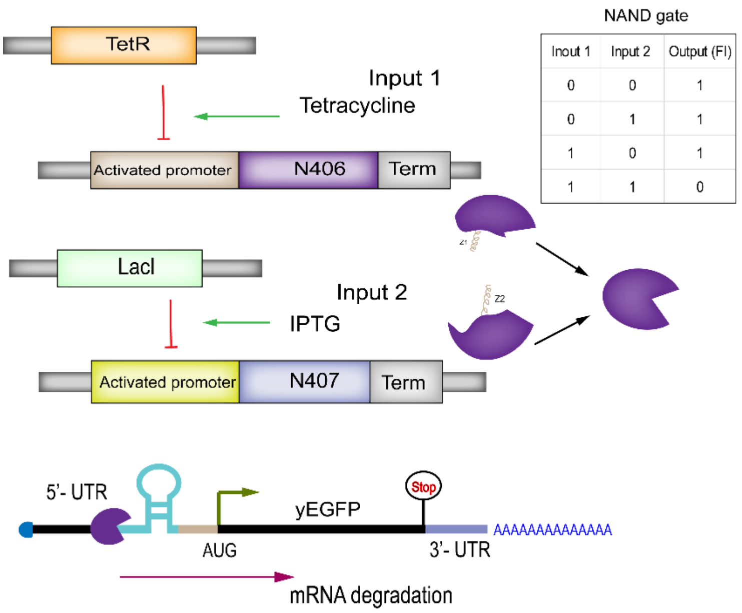

Figure S7. Potential usage of dCasP:pre-crRNA mRNA degradation tool: a complex NAND Boolean gate scheme responding to tetracycline and IPTG.

Materials and Methods

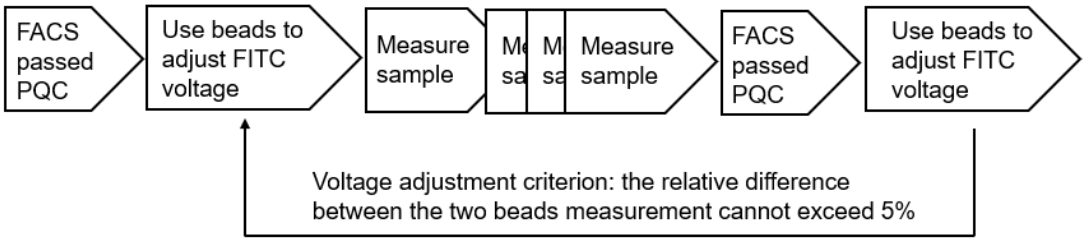

Figure SMM1. Performance quality control and usage of fluorescence beads to determine the status of the FACS machine and the experiment reliability.

OFF-TARGET Effects Analysis

In the pre-crRNA sequences placed either in the 5’- or 3’ UTR of the target mRNA, we used overall nine different spacers (see Table SO1).

Number	Sequence	Length (nt)
1	GAGAAGTCATTTAATAAGGCCACTGTTAAAA	31
2	GCTACTATTCCTGTGCCTTCAGATAATTCA	30
3	GTCTAGAGCCTTTTGTATTAGTAGCCG	27
4	TAGCGATTTATGAAGGTCATTTTTTT	26
5	AGATTAAAAGGTAATTCTATCTTGTTGAG	29
6	TACCTAGTAGATACGCTTACTGATAACAA	29
7	AAACTTTCATTTATGATATAAAGTTTTTT	29
8	TCAAAAGGCAAGAGAGACGGAAATAAATGGAC	32
9	TTGTTTGATTGCTTGCATTGA	21

Table SO1. Spacers used in the pre-crRNA sequences.

They were taken from bacterial genomes. We verified *in silico* that none of these spacers represented a target on the yeast genome for the dCasP:crRNA complex. To this aim we used the web-server Cas-OFFinder (http://www.rgenome.net/cas-offinder/). For each spacer, we checked both the full sequence (truncated at the 25th nucleotide, where necessary, following the software requirements) and the minimal one (the first 15 nucleotides). We set “Mismatch Number”, “DNA Bulge Size”, and “RNA Bulge Size” equal to 0 (the default value). As for the “Target Genome”, we chose, under “Others”, “*Saccharomyces cerevisiae* s288c”. On every spacer, we run the analysis with three different “PAM Types”: “AsCpf1 from *Acidaminococcus* or LbCpf1 from *Lachnospiraceae*: 5'-TTTV-3' (V = G or C or A)"; “FnCpf1 from *Francisella*: 5'-TTN-3'”; and “FnCpf1 from *Francisella*: 5'-KYTV-3'”.

Afterwards, we selected two circuits for transcriptomic analysis. They corresponded to the strains: byMM1501, containing 9xFn_Rs on the 3’ UTR, and byMM1592, hosting 1xFn_Rs on the 5’ UTR. More in detail, the two strain were constructed as:

- byMM1501: pMM608 + byMM1494 (LEU2-pCYC1min-yEGFP-**9xFn_Rs**-CYC1t and URA3-pGPD-dLbCas12a-CYC1t);
- byMM1592: pMM1450 + byMM1565 (LEU2-pTEF2-**1xFn_Rs**-yEGFP-CYC1t and URA3-pGPD-dFnCas12a-NES-CYC1t).

The four strains were analyzed by Novogene Co., Beijing, (China) (Transcriptome sequencing with Illumina Hiseq-PE150-see also Supplementary Material-transcriptome). Results are shown in Figure SO1.

A

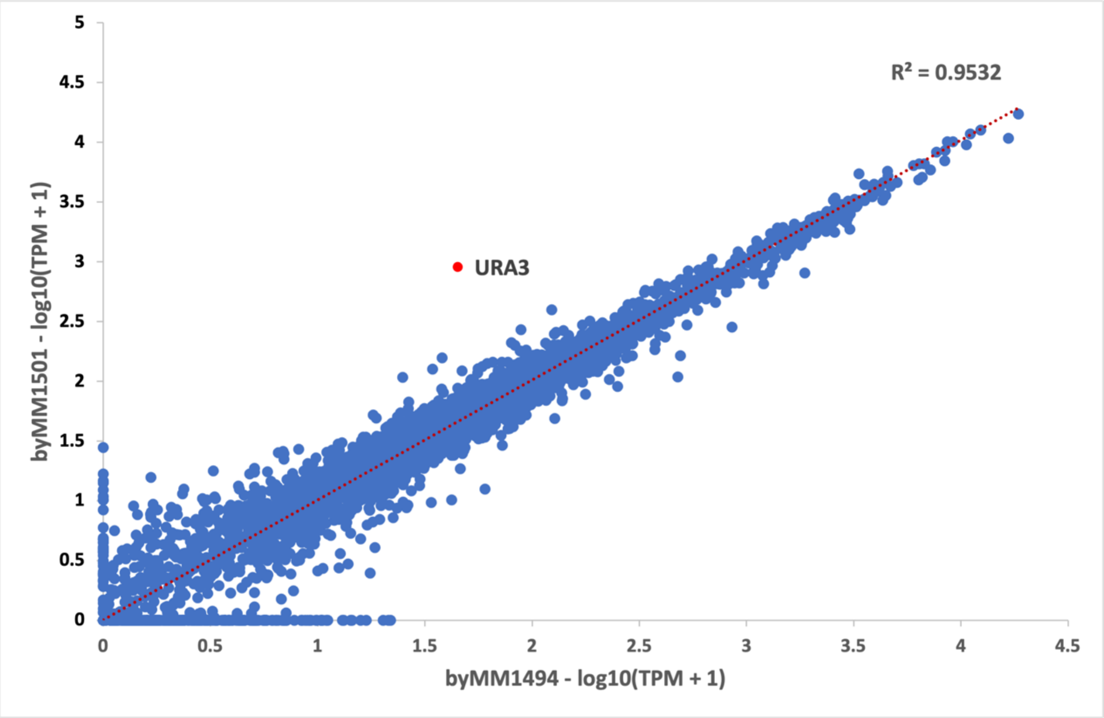

B

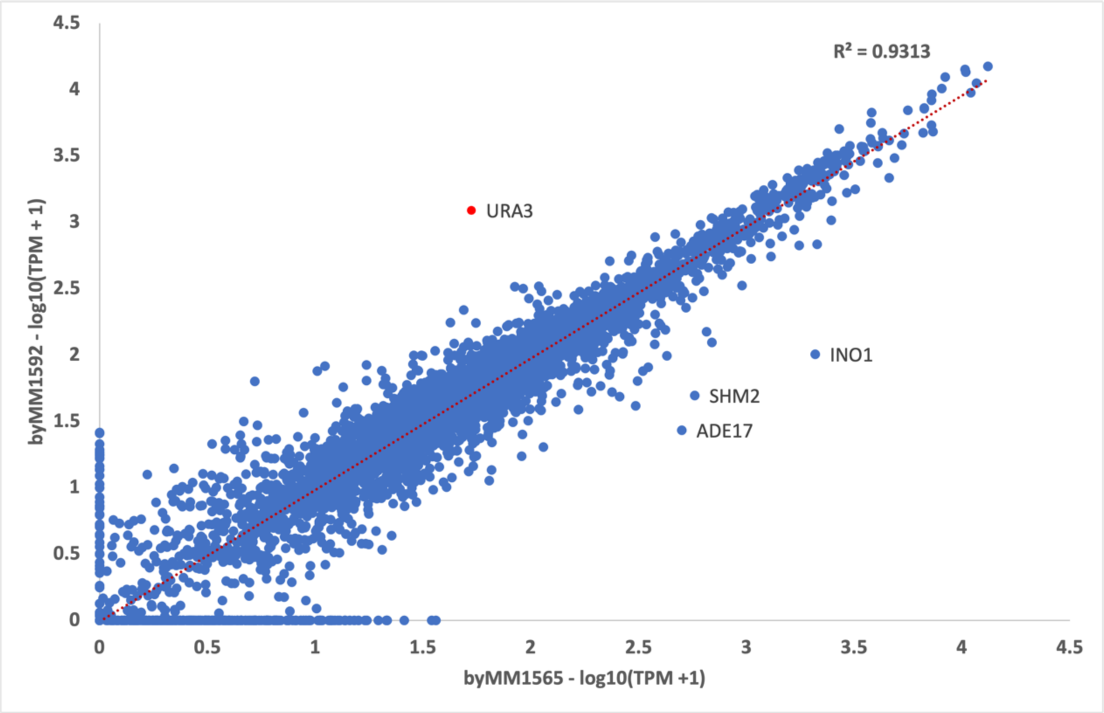

Figure SO1. Gene expression levels in $\log_{10}$ of transcripts per million. (A) byMM1501 versus byMM1494. The value of R^2 (0.95) is sufficiently high to claim that most of the genes in the two strains are expressed in equal amount. (B) byMM1592 versus byMM1562. Even though a single spacer is used, the R^2 is lower than in (A), with three genes

(INO1, SHM2, and ADE17) that appear downregulated in byMM1592. We think, however, that this result is more likely due to imprecision in the measurements rather than to OFF-target effects. The URA3 gene is mutated (*ura3-52* mutation), into byMM1494 and byMM1565. Indeed, its expression is higher in both byMM1501 and byMM1592 strains, that contain a functional URA3 gene because of the integration of the plasmids bringing dCasP. Each analysis has been carried out only once.

On the whole, we think that the transcriptomes of the four strains here taken into account confirm the outcome of the *in silico* analysis, i.e., no OFF-target effects are present that could unbalance the cell metabolism or even lead yeast cells to death.
